# Upper esophageal sphincter mechanical states analysis: a novel methodology to describe UES relaxation and opening

**DOI:** 10.3389/fnsys.2014.00241

**Published:** 2015-01-07

**Authors:** Taher I. Omari, Lukasz Wiklendt, Philip Dinning, Marcello Costa, Nathalie Rommel, Charles Cock

**Affiliations:** ^1^Department of Human Physiology, School of Medicine, Flinders UniversityAdelaide, SA, Australia; ^2^Department of Gastroenterology and Hepatology, Flinders Medical Centre and School of Medicine, Flinders UniversityAdelaide, SA, Australia; ^3^Translational Research Center for Gastrointestinal Diseases, University of LeuvenLeuven, Belgium

**Keywords:** swallow, dysphagia, pressure, impedance, neural pathways, upper esophageal sphincter, cricopharyngeus muscle, suprahyoid muscles

## Abstract

The swallowing muscles that influence upper esophageal sphincter (UES) opening are centrally controlled and modulated by sensory information. Activation of neural inputs to these muscles, the intrinsic cricopharyngeus muscle and extrinsic suprahyoid muscles, results in their contraction or relaxation, which changes the diameter of the lumen, alters the intraluminal pressure and ultimately inhibits or promotes flow of content. This relationship that exists between the changes in diameter and concurrent changes in intraluminal pressure has been used previously to calculate the “mechanical states” of the muscle; that is when the muscles are passively or actively, relaxing or contracting. Diseases that alter the neural pathways to these muscles can result in weakening the muscle contractility and/or decreasing the muscle compliance, all of which can cause dysphagia. Detecting these changes in the mechanical state of the muscle is difficult and as the current interpretation of UES motility is based largely upon pressure measurement (manometry), subtle changes in the muscle function during swallow can be missed. We hypothesized that quantification of mechanical states of the UES and the pressure-diameter properties that define them, would allow objective characterization of the mechanisms that govern the timing and extent of UES opening during swallowing. To achieve this we initially analyzed swallows captured by simultaneous videofluoroscopy and UES pressure with impedance recording. From these data we demonstrated that intraluminal impedance measurements could be used to determine changes in the internal diameter of the lumen when compared to videofluoroscopy. Then using a database of pressure-impedance studies, recorded from young and aged healthy controls and patients with motor neuron disease, we calculated the UES mechanical states in relation to a standardized swallowed bolus volume, normal aging and dysphagia pathology. Our results indicated that eight different mechanical states were almost always seen during healthy swallowing and some of these calculated changes in muscle function were consistent with the known neurally dependent phasic discharge patterns of cricopharyngeus muscle activity during swallowing. Clearly defined changes in the mechanical states were observed in motor neuron disease when compared to age matched healthy controls. Our data indicate that mechanical state predictions were simple to apply and revealed patterns consistent with the known neural inputs activating the different muscles during swallowing.

## Introduction

The upper esophageal sphincter (UES) is a region of high pressure located at the juncture between the pharynx and esophagus. The UES is a gate-keeper which prevents breathed-in air from entering the esophagus and guards against aspiration of gastric reflux. The UES high pressure zone must also relax and the lumen must open to allow unimpeded passage of swallowed food. The neural inputs to the swallowing muscles that influence UES pressure relaxation and luminal opening are controlled by central circuits and these are modulated by sensory information. Activation of these neural inputs results in a complex sequence of changes in the striated muscles. These changes could see the muscles contract or relax, which in turn influences the intraluminal pressures and diameters ultimately determining the propulsion of swallowed food. Of most relevance to the UES are the intrinsic cricopharyngeus muscle and the extrinsic suprahyoid muscles which are mechanically coupled to the UES.

Changes in the mechanical states of the UES, such as failed opening, can result in dysphagia. Diagnosis of UES opening dysfunction is challenging yet critical because accurate diagnosis is predictive for therapeutic outcomes. Reliable interpretation of UES motor patterns is mostly based on pressure measurement (manometry), however determining normal or abnormal UES opening requires considerable interpretive expertise (Hila et al., 2001; Bhatia and Shah, 2013). This is because the typical markers of UES flow resistance (i.e., failed or incomplete opening), are residual pressure and the intrabolus pressure, and these are contingent upon pressure generation from above the UES through sequential lingual propulsion and contraction of pharyngeal constrictors (Ali et al., 1997; Williams et al., 2002; Pal et al., 2003). As such, UES residual pressure and intrabolus pressure gradient are less predictive in patients with neuromuscular conditions due to concomitant poor lingual bolus control and pharyngeal contractile weakness (Cook et al., 1989; Williams et al., 2002).

Adding to the difficulties in diagnosing UES dysfunction, the majority of conditions affecting oropharyngeal swallowing occur in the aged population where neuromuscular incoordination and weakness are more prevalent (Cook, 2009). To further complicate matters a clear distinction exists between manometrically measured UES “relaxation” and radiologically observed UES “opening” (Cook et al., 1989). Manometrically determined relaxation does not equate to the extent of UES opening during flow. A “non-relaxing” UES suggests failure of cessation of cricopharyngeus (CP) muscle excitation by spinal motor neurons, however the degree to which this type of dysfunction may limit UES opening is dependent upon whether the pharyngeal contractile front, driving bolus propulsion from above, is preserved or fragmented, as well as whether external traction forces being applied by the suprahyoid muscles to open the UES are sufficiently strong and appropriately timed (Williams et al., 2002). In healthy subjects and patients with an intact pharyngeal swallow, the volume of the bolus being propelled is also a major contributor to the timing and extent of UES opening (Kahrilas et al., 1988; Cook et al., 1989; Williams et al., 2002).

In summary, manometry alone gives an incomplete description of UES function during swallowing. Although radiological studies have been correlated with manometry and attempts have been made to establish the biomechanics of UES opening and failure of opening (Williams et al., 2002; Pal et al., 2003), these studies have not resulted in simple procedures enabling the reliable analysis and diagnoses of UES motor dysfunctions.

To improve our ability to detect physiological differences in UES function in health and disease, we need to be able to detect subtle changes in UES opening and closing. In this paper we will describe a novel technique, adapted from laboratory based *ex vivo* studies of gut peristalsis (Costa et al., 2013), which allows assessment of the major components of UES function by relating real-time changes in the diameter of the lumen with the corresponding changes in intraluminal pressure recorded at the same location and time. Through defining this relationship between changes in diameter and pressure, we can define the mechanical state of the muscle (refer to Box 1).

Box 1 **The UES mechanical states concept.**

**Figure 1:**
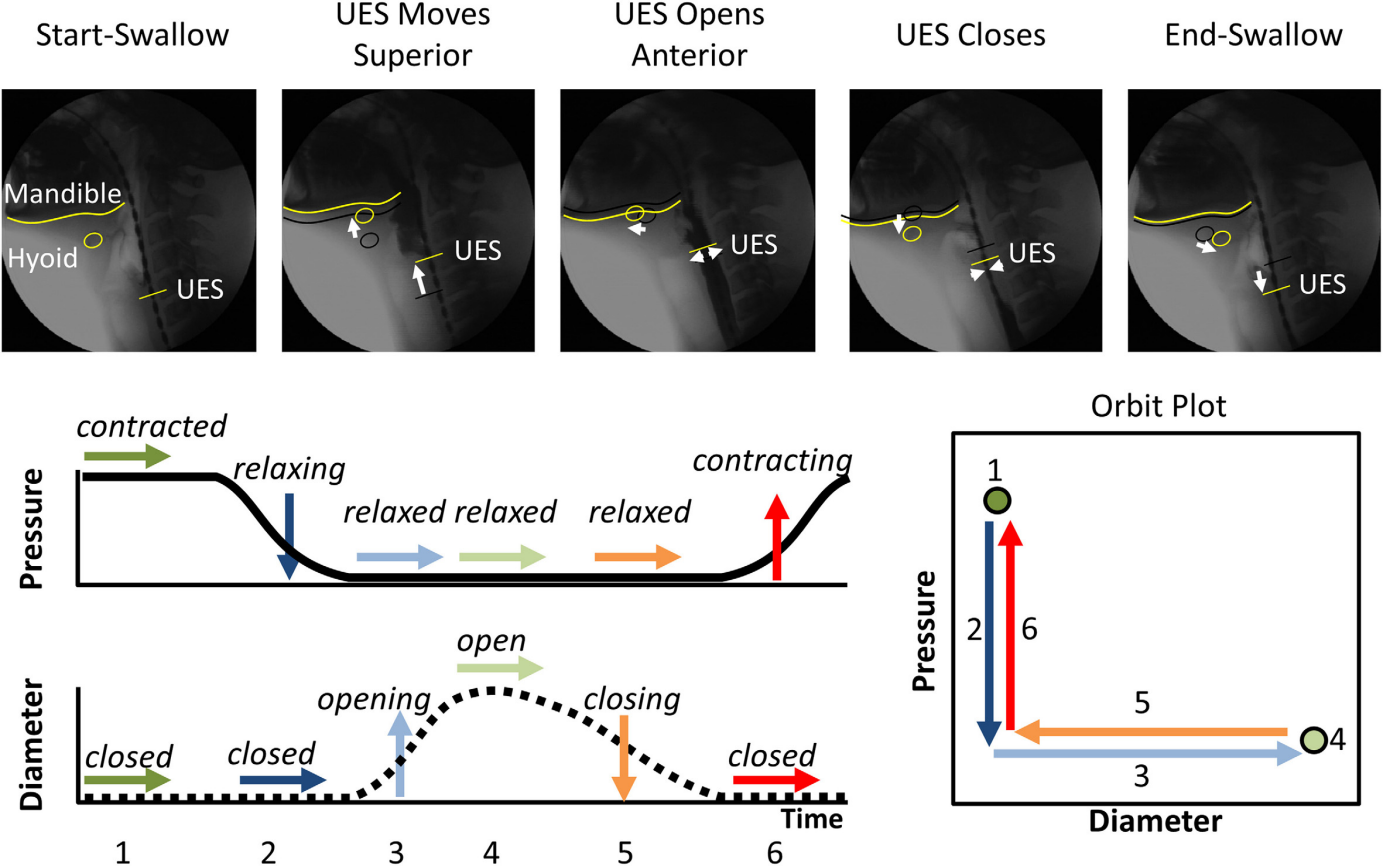
**The typical pattern of pressure and diameter change that the UES undergoes during normal swallowing**. Videofluoroscopy images show the different phases of swallow. The position of the inferior edge of the mandible, the hyoid bone and the UES proximal margin are outlined in yellow in each frame. White arrows show movement of the relevant anatomical structures relative to their position from previous frame (shown as black shadow). A typical UES pressure profile is shown with arrows indicating periods when pressure is static (→), decreasing (↓) or increasing (↑). A typical diameter profile is shown with arrows indicating periods when the diameter is static (→), increasing (↑) or decreasing (↓). When the pressure and diameter data are interpreted together, different mechanical states can be defined based on the direction of pressure change and in relation to whether the lumen is open, closed or changing in diameter. The relationship of diameter vs. pressure over time can also be visualized by way of an “Orbit” plot. The mechanical states numbered 1–6 typify the normal sequence of UES contractility and UES opening during swallowing. Note: Nomenclature describing the states shown is provided in Figure 2.

Pressure changes occurring within the UES high pressure zone during swallowing of a bolus material can be measured with an indwelling catheter and diameter changes of the UES lumen can be measured using videofluoroscopy imaging of barium-containing bolus material. UES pressure is predominantly generated by the cricopharyngeus muscle (CP) and, during normal swallowing, the measured UES pressure fluctuates in concert with neural activation and deactivation of the CP. The hyoid bone (as shown Figure 1) is an anatomical structure mechanically coupled to the UES. The UES opens anteriorly predominantly due to neurally mediated contraction of the suprahyoid muscles which are attached to the hyoid bone.

**Figure 2:**
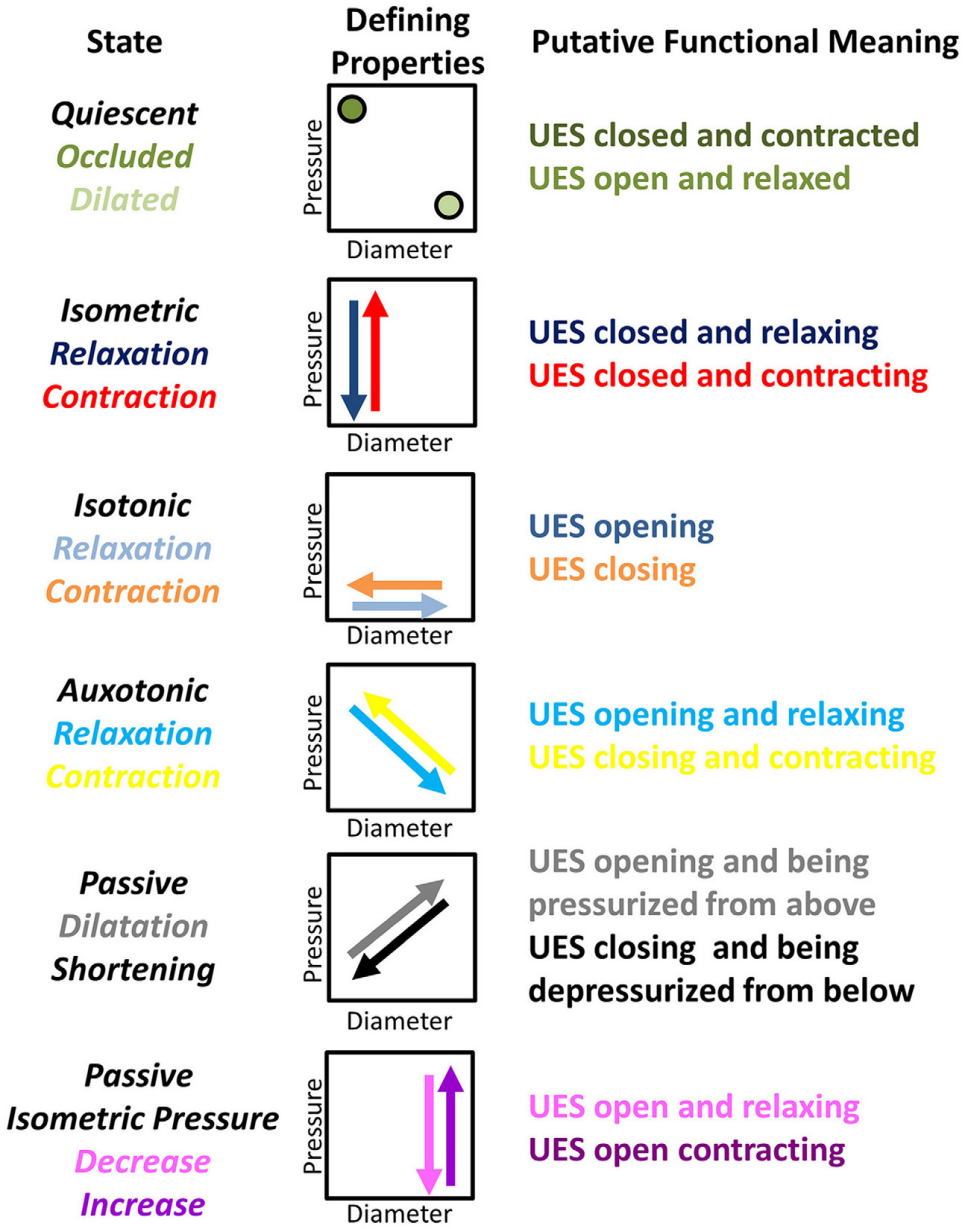
**Nomenclature and defining properties of 12 possible UES Mechanical States previously described in relation to propulsive peristalsis of the lower gut (Costa et al., 2013)**. The putative functional meaning when applied to the UES is also provided.

By examining the relationships that exist between changes in diameter and the corresponding changes in pressure, recorded at the same point in space and time, the mechanical states of the muscle can be determined (Costa et al., 2013). These mechanical states, predict when the muscle is actively contracting or relaxing during periods of luminal occlusion or distension. During a human swallow these changes in diameter and pressure can be seen in Figure 1. The relationship between diameter and pressure over time can also be visualized by way of an “Orbit” plot (Figure 1). Nomenclature describing the UES mechanical states has been provided in Figure 2 (refer also to **Box 2** regarding specific terminology applied in the nomenclature). Previous studies examining *ex vivo* peristalsis in the lower gut have defined 12 possible mechanical states (8 active and 4 passive) (Costa et al., 2013; Wiklendt et al., 2013; Dinning et al., 2014) and these have been detailed in **Box 2**, Figure 2. In the human UES at least six of these mechanical states can be used to typify the normal sequence of UES contractility and UES opening during swallow (see colored arrows in Figure 1). Based on the current understanding of UES relaxation and opening mechanisms a putative meaning for the different mechanical states can be suggested (**Box 2**, Figure 2). Each state being the net result of passive elasticity or compliance of the muscle, active contractile states of the muscle (driven by neurogenic pathways) and extrinsic tractional forces.

We sought to apply this new method to *in vivo* recordings of UES relaxation and opening during bolus swallowing. The swallowing muscles of the pharyngo-esophageal segment are striated and therefore only contract via inputs from spinal motor neurons. To record this neural input traditionally involves EMG recordings. However, such recordings are usually limited to a single site. For *in vivo* human recordings, luminal manometry offers greater spatial resolution and is simpler and easier to apply than EMG. Therefore, manometry offers a distinct advantage over EMG provided that the information gathered on motility of the UES can give similar insights. By applying the methodology used to calculate the mechanical states we can theoretically determine in real-time when the muscle is actively contracting or relaxing in response to neural inputs (Costa et al., 2013).

Calculation of the mechanical states also requires accurate measurement of diameter. To approximate changes in diameter in a real-time and *in vivo* situation we have used intraluminal impedance, which is recorded in parallel with the manometry. In both *ex vivo* (Costa et al., 2013) and *in vivo* recordings (Omari et al., 2012; Kim et al., 2014) it has been shown that impedance can be used to estimate changes in diameter in association with bolus movement, thus negating the need for radiology. In this paper we provide further validation of this technique.

### Hypothesis

We hypothesize that quantification of the different mechanical states of the UES and the mechanical properties that define them, namely pressure change and diameter change, may allow objective characterization of mechanisms that govern the timing and extent of UES relaxation and UES opening during normal and disordered swallowing. In order to address our hypothesis we performed investigations to both validate the methodology and to apply it to a “real world” dysphagia patient scenario.

Our study had four aims. Firstly, we aimed to use combined videofluoroscopy and UES manometry to characterize the mechanical state profile occurring within the UES region based on the inter-relationships of UES diameter (recorded by X-ray) and pressure change over time. Secondly, we aimed to determine if UES mechanical states can also be predicted using intraluminal impedance measurement to estimate diameter change in place of videofluoroscopy. In order to achieve this we sought to characterize the relationship between diameter and intraluminal impedance, hence enabling non-radiological application of the technique. Thirdly, we aimed to define a pressure-impedance based model that would also accommodate the superior movement of the UES high pressure zone during the swallow. Finally, following completion of the validation studies above, we aimed to apply the optimized pressure-impedance model to a database of previously acquired studies. Specifically, we examined changes in UES mechanical states in relation to bolus volume, normal aging and in patients with dysphagia symptoms due to motor neuron disease.

## Materials and methods

### Validation studies

#### Study procedure

The diameter, impedance and pressure relationships were determined for the UES by use of continuous videofluoroscopy imaging in conjunction with intraluminal impedance and pressure measurement using an indwelling catheter placed across the UES. Combined simultaneous videofluoroscopy and pressure-impedance studies of pharyngeal swallows from five healthy controls (3 male; 24–45 yrs, mean 38) were analyzed. Each subject was intubated with a 3.2 mm diameter solid state pressure and impedance catheter incorporating 25 1 cm-spaced pressure sensors and 12 adjoining impedance segments, each of 2 cm length (Unisensor USA Inc, Portsmouth, NH). Subjects were intubated after topical anesthesia (lignocaine spray) and the catheter was positioned with sensors straddling the entire pharyngo-esophageal segment (velo-pharynx to proximal esophagus). Following accommodation to the catheter, a brief fluoroscopy screen ensured the most proximal sensor of the pressure-impedance array was in view. With the subject sitting upright and in the head neural position, three swallows of 10 ml liquid barium contrast material (MicropaqueH™ containing 1% NaCl to make the bolus conductive) were captured simultaneously by continuous videofluoroscopy (25 frames/s) and the pressure-impedance acquisition system (data sampling at 20 Hz, Solar GI system, MMS, The Netherlands).

#### Measurement of UES diameter change during swallow

Our initial analysis and prediction of UES mechanical states was based on pressure recordings and diameter measurements within a 2 cm region located along the pressure-impedance array of the catheter and identified by a single impedance segment optimally located such that it was observed on videofluoroscopy to remain within the region of the UES during the swallow. Pressure measurements were taken based on the pressure sensor located at the mid-point of the segment, which we called the *Reference Impedance Segment* because it was subsequently used to establish the relationship of diameter with impedance (see below). Diameter was measured at three points along the reference segment based on the simultaneously acquired videofluoroscopy images. Whilst we recognize that UES moves independently of the catheter, use of a fixed location for these initial validation studies allowed us to avoid the need to correct for these complex movements.

**Figure 3:**
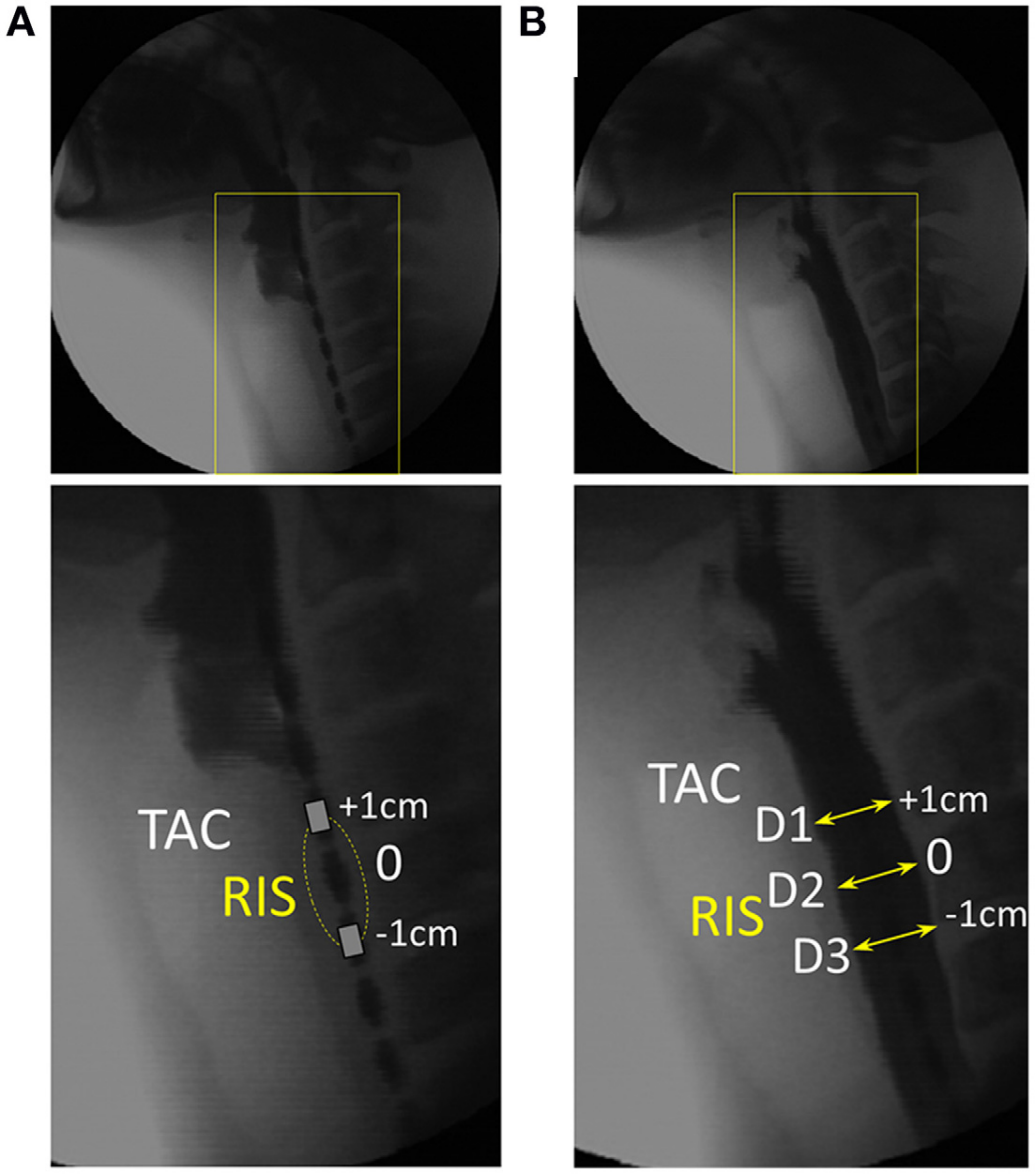
**Videofluoroscopy images showing how luminal diameter was precisely measured over time**. For this example 10 ml liquid swallow the approximate position of the UES is identified through visualization of the tracheal air column (TAC). **(A)** Image immediately preceding UES penetration by liquid contrast. The most optimally located reference impedance segment (RIS) is adjacent to the TAC. **(B)** Image showing the barium column at maximum diameter and surrounding the RIS. Diameter was measured at the axial center (D2) and at both the proximal and distal ring electrodes (D1, D3) demarcating the margins of the reference impedance segment. Sequential measurements were performed from entry to exit of all of the barium within the RIS.

For each of 15 captured swallow sequences, the timing of first superior movement of the hyoid bone was used to mark the timing of swallow onset. The position of the proximal UES was identified by visualization of the tracheal air column. The video was then advanced frame-by-frame to observe superior and anterior laryngeal movement until the time point immediately preceding UES penetration by liquid contrast (Figure 3A). The most optimally located impedance segment (ring electrode pair spaced at 2 cm apart) was then determined as the segment which was most closely adjacent to the UES, but also inferior of the air-tissue interface ensuring that no part of the impedance segment was in the pharyngeal air space. The video was then advanced frame by frame (0.04 s steps) and the diameter of the barium column measured for each frame at three locations along the reference segment. These were the proximal electrode ring, the pressure sensor at midpoint of the segment and the distal electrode ring (Figure 3B).

**Figure 4:**
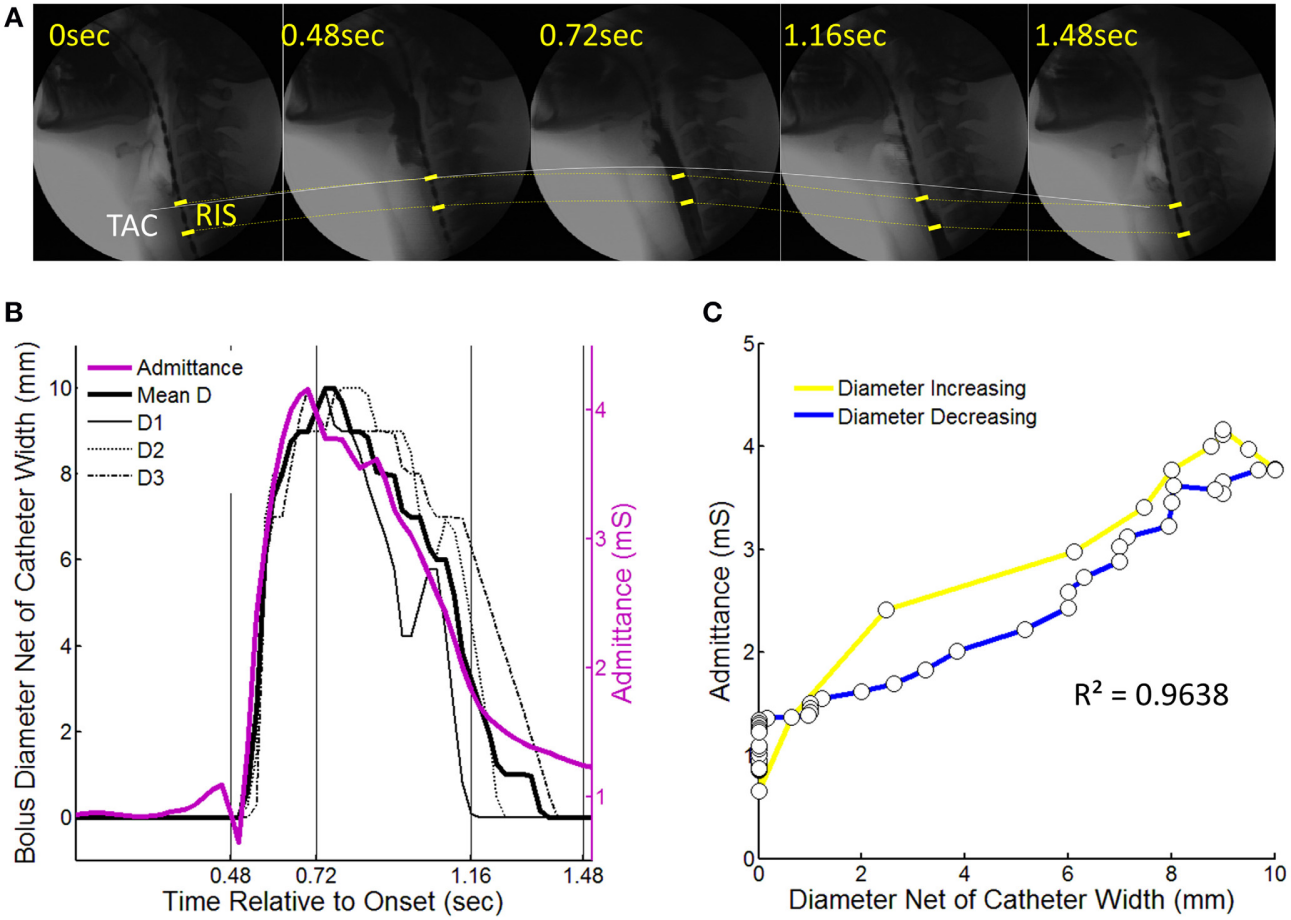
**An example of a 10 ml liquid swallow showing the temporal correlation of luminal diameter and intraluminal admittance at the level of the reference impedance segment (RIS)**. **(A)** Sequential images from swallow onset (laryngeal elevation) showing barium passing through the RIS. Dotted lines chart the trajectory of movement of the tracheal air column (TAC, white line) and the RIS (yellow dotted line). Note decoupling of the TAC and proximal margin of the RIS at time points 0.72 and 1.16 s after swallow onset. **(B)** Diameter of the barium contrast column and admittance measured at the level of RIS over time from swallow onset. Individual diameter curves are shown for locations D1–D3 (per Figure 3) as well as the mean diameter. **(C)** Time correlation of diameter and admittance with colors indicating whether samples correspond to when the lumen was increasing (yellow) or decreasing (blue) in diameter.

Sequential diameter measurements were performed until all of the barium had passed through the reference impedance segment (Figure 4A). The catheter electrode rings produced a discernible radiological shadow. Therefore, even though the lumen contained contrast and there was the inevitable decoupling of the axial positions of the tracheal air column and the reference segment due to further superior laryngeal movement, we were still able to accurately measure the diameters at very precise locations along the pressure-impedance catheter at all times (Figure 4A). All measurements were calibrated for magnification by using the known distance between visible adjacent electrodes. Diameters were expressed net of the width of the indwelling catheter (~3 mm) such that the lumen fully closed on the catheter was defined as having a diameter of 0 mm (rather than 3 mm). The three separate diameter measurements (D1–D3 Figure 3B) taken over consecutive video frames were averaged for each time point to produce a single value of diameter for the reference segment over time. The diameter dataset was then smoothed by resampling from 25 to 40 Hz using a piece-wise cubic Hermite interpolation method. The corresponding midpoint pressure data and impedance data for the reference segment were then exported from the acquisition system in comma-separated value text-file format. These data were smoothed by interpolation to match the diameter dataset as previously described.

#### Definition of UES mechanical states: diameter-pressure method

Refer to Figure 2 and Box 2 regarding our use of specific terminology to describe UES mechanical states.

Box 2 **An explanation of terminology used to describe UES mechanical states.**

As the UES is comprised of striated muscle, the physiology governing the smooth muscle gut peristalsis is not directly comparable. Furthermore, UES *relaxation* is physiologically and biomechanically distinct from UES *opening*. Nevertheless, we considered that the previously described 12 mechanical states characterized in relation to peristalsis (Costa et al., 2013) should also be observable during transit of a swallowed bolus through the UES (Figure 2).

For this pilot investigation, and first description of the methodology in relation to the UES, we have purposefully maintained the descriptive terminology of mechanical states applied in our previous study of colonic peristalsis *ex vivo* (Costa et al., 2013; Wiklendt et al., 2013; Dinning et al., 2014). We acknowledge that, in some instances, our use of terms such as *“relaxation,” “contraction,”* and *“quiescence”* may not be considered functionally appropriate when used in conjunction with the physiological mechanisms that govern the mechanical events we are seeking to measure. One example is the definition of “Occluded Quiescence” which is applied when the UES is closed and the pressure is neither increasing nor decreasing. Under basal conditions, it has been shown that UES pressure is generated by myogenic and neurogenic mechanisms and combined with force due to elasticity of the surrounding tissues (Levitt et al., 1965; Asoh and Goyal, 1978; Lang et al., 1991; Medda et al., 1997). It could therefore be argued that this state can be defined as a separate Isometric state. Another example is use of the term “Relaxation” in relation to mechanical states when the UES is opening and “Contraction” when the UES is closing. It can be argued that the opposite terminology should be used because the UES opens when anterior traction is applied by contraction of suprahyoid muscles. However, because the approach is both new and complex, we have decided to maintain the original generic terminology and will consider more functionally appropriate terms in future studies.

Using the temporally aligned and synchronized diameter and pressure data, samples for each of the 15 liquid swallows was consecutively assigned a mechanical state based on the direction of contraction or relaxation and in relation to whether the lumen was in an occluded or distended state. The 12 possible mechanical states, as defined in Figure 2, were determined using the derivatives of diameter and pressure with respect to time. A minimum diameter of ≤1 mm defined an occluded state and a rate of diameter change in excess of ±2 mm/s defined whether the diameter was increasing or decreasing. Based on a separate analysis of the derivative of normal UES resting pressure fluctuations with respect to time, criteria of +250 mmHg/s or −150 mmHg/s defined the thresholds which determined whether pressure was considered to be increasing or decreasing above or below the level of baseline activity.

#### Time correlation of UES diameter and UES impedance

As previously described we measured diameter and impedance at a single 2 cm reference impedance segment optimally located within the region of the UES. To account for known non-linearity of the impedance-diameter relationship (Kim et al., 2014) the impedance values were converted to the inverse product of impedance (1/impedance) called *“Admittance”* and expressed in siemens (S) or millisiemens (mS). The extent of temporal correlation between UES diameter and UES admittance was then determined for each individual swallow (Figures 4B,C) and averaged for all swallows.

#### Definition of UES mechanical states: admittance-pressure method

Refer to Figure 2 and Box 2 regarding our use of specific terminology to describe UES mechanical states.

Using the simultaneous admittance and pressure data recorded for the reference segment (as described above) samples for each of the 15 liquid swallows was consecutively assigned a mechanical state based on the direction of contraction or relaxation and in relation whether the lumen was in an occluded or distended state. The 12 mechanical states, as defined in Figure 2, were determined using the derivatives of admittance and pressure with respect to time. Having established a model for determining UES mechanical states based on diameter as the gold standard, optimal admittance criteria were defined following an iterative process (data not shown). Admittance of 5–15 mS defined an occluded state. A rate of admittance change above +10 mS/s or below −1.5 mS/s defined whether the diameter was increasing or decreasing. As for the diameter-pressure method, a pressure change threshold of >250 mmHg/s was used to define pressure increasing above baseline tone and a threshold of <−150 mmHg/s used to define pressure decreasing below basal tone.

#### Accommodation of superior movement of the larynx during swallow

Using the impedance-pressure criteria described above, the model was modified to accommodate the 2 cm or more elevation of the sphincter that occurs before complete UES relaxation (Kahrilas et al., 1988). This was done by measuring all axial pressures within the limits of high pressure zone over time. The location of maximum axial pressure demonstrates the superior movement of the UES (Ghosh et al., 2006).

### Studies in healthy controls and motor neuron disease patients

Following development of the non-radiological admittance-based method as described above, the UES mechanical states model was applied to an independent database of previously acquired impedance-pressure recordings. This additional analysis was performed in order to characterize the effect of liquid bolus volume on mechanical states, as well as to characterize what happens to the mechanical states when the motor pathways controlling the swallowing muscles are impaired due to neurological pathology.

Swallows from 67 healthy control subjects age 20–91 years and 11 patients with motor neuron disease (MND) aged 58–91 years were analyzed. Studies were performed as previously described using solid state pressure and impedance catheters of 3.2 mm diameter (Unisensor USA Inc, Portsmouth, NH). Two catheter configurations were used: Catheter 1; incorporating 25 1 cm-spaced pressure sensors and 12 adjoining 2 cm impedance segments (All Controls and four MND Patients), or Catheter 2; incorporating 36 1 cm-spaced pressure sensors and 16 adjoining 2 cm impedance segments (seven MND patients).

Control subjects were recruited by community advertisement and excluded if they had evidence of any pharyngeal swallowing difficulties, symptoms suggestive of a motility disorder, upper gastrointestinal conditions including gastroesophageal reflux disease, diabetes mellitus, a previous history of gastrointestinal surgery or were taking any medications known to affect GI motility.

The cohort of patients with motor neuron disease included in the study had been referred to the swallowing disorders clinic for assessment of swallowing dysfunction by a gastroenterologist and expert swallowing speech pathologist. Their functional status varied from requiring occasional assistance to normal activity with effort [Score of 60–80 on the *Australia-modified Karnofsky Performance status scale* Abernethy et al., 2005]. Percutaneous endoscopic gastrostomy (PEG) placements were performed in eight patients within 6 months following measurement.

All subjects and patients underwent the same manometric procedure as previously described. After intubation and a 10 min accommodation period, subjects received five 5 ml boluses followed by five 10 ml boluses of saline (0.9% NaCl). Boluses were administered at >20 s intervals to the mouth via a syringe and subjects asked to swallow on command (i.e., cued volitional swallowing).

Patients underwent a videofluoroscopic study of their swallowing. However, this was not performed at the same time as the manometric procedure. Two of the patients in this cohort had overt pulmonary aspiration on swallowing of liquids during videofluoroscopic swallow studies, whilst the remainder had no evidence of pulmonary aspiration. Six patients had evidence of significant pharyngeal residue (>50% vallecular/piriform sinus residue). Radiology was not performed in Controls.

In addition to the UES mechanical states analysis, each bolus swallow was also analyzed to determine the pre- and post-relaxation maximum UES pressure, the UES nadir pressure during relaxation and 0.2 s UES integrated relaxation pressure (Weijenborg et al., 2014).

### Statistical analysis

Regression r^2^ was used to assess strength of temporal correlation between diameter and admittance. Per-volume averages were calculated for each subject and then compared between Control and Patient groups. These data were not normally distributed and therefore presented as Median [Interquartile Range]. Comparisons were performed using Wilcoxon Signed Rank Test for paired data; Kruskal-Wallis One Way Analysis of Variance on Ranks with Dunn's Method used for pairwise multiple comparisons within grouped data and Spearman-Rank correlation. Prognostic value was assessed using the area under the Receiver Operator Curve (AUC). A *p* < 0.05 was considered statistically significant.

## Results

### Validation studies

#### Temporal correlation of UES diameter and impedance

**Figure 5:**
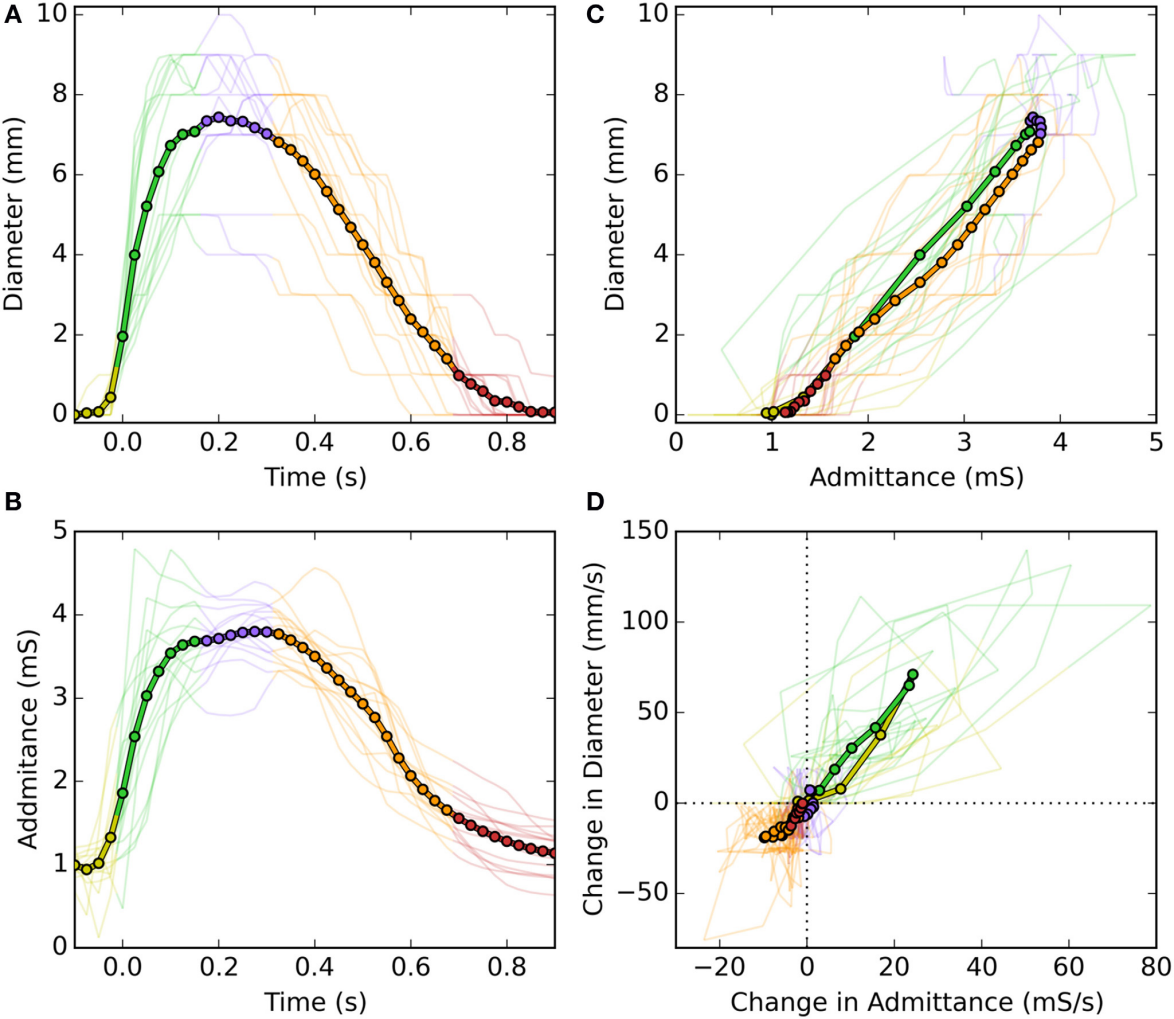
**Temporal correlations of UES diameter and UES admittance during a swallow**. **(A)** UES diameter over time of a swallow. **(B)** UES admittance over the same period of time. **(C)** Time correlations of diameter vs. admittance. **(D)** Time correlation of the time derivatives of diameter vs. admittance. In each graph fine lines show data from each swallow and the thick line shows the average of all swallows. Colors indicate the different stages of luminal opening (yellow, pre-opening; green, opening; blue, open; orange, closing; red, closed).

Following careful time and space synchronization and alignment, we observed a strong linear time correlation between UES diameter and UES admittance. For individual swallows the level of correlation ranged from regression *r*^2^ = 0.79–0.98 (mean 0.93). As shown is Figure 4C, some hysteresis was typically observed in the time correlation whereby the level of admittance differed during the diameter increasing vs. decreasing phases of the swallow. Whilst a consistent pattern of hysteresis was not apparent across all swallows, the admittance trended higher during the closing phase and admittance was also elevated from the pre-swallow baseline at the time of radiologically observed luminal closure onto the catheter (apparent in Figure 4B). Across all analyzed swallows the averaged data for diameter and admittance over time produced an almost perfect correlation and similar slope for the diameter increasing and decreasing phases of UES opening (Figures 5A–C). The rate of diameter change and admittance change (i.e., the derivative of the time correlation) was much faster when the UES lumen was opening, compared to when the lumen was closing (Figure 5D).

#### Predictions of UES mechanical states

**Figure 6:**
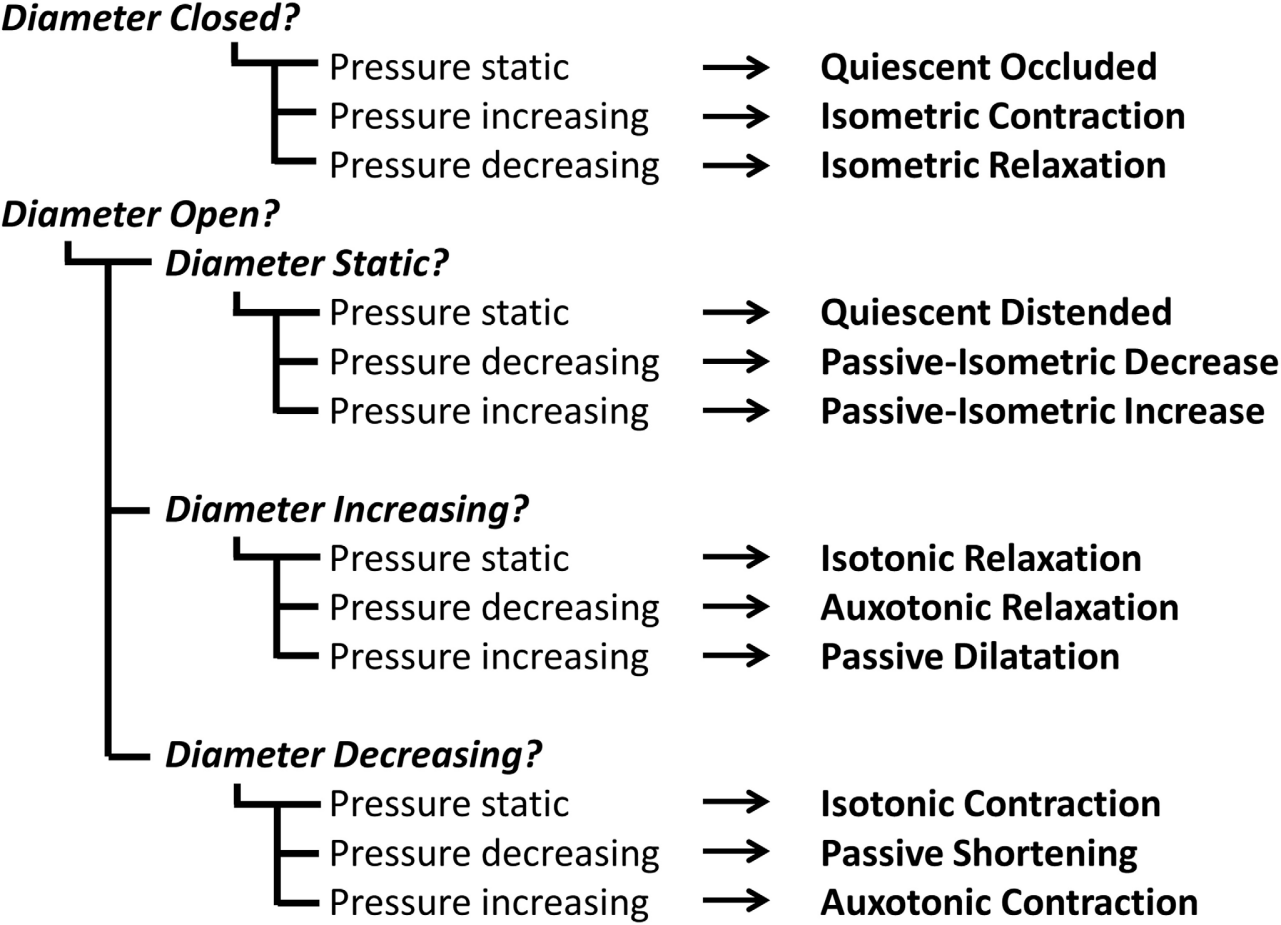
**Decision tree applied to define UES mechanical states**.

**Figure 7:**
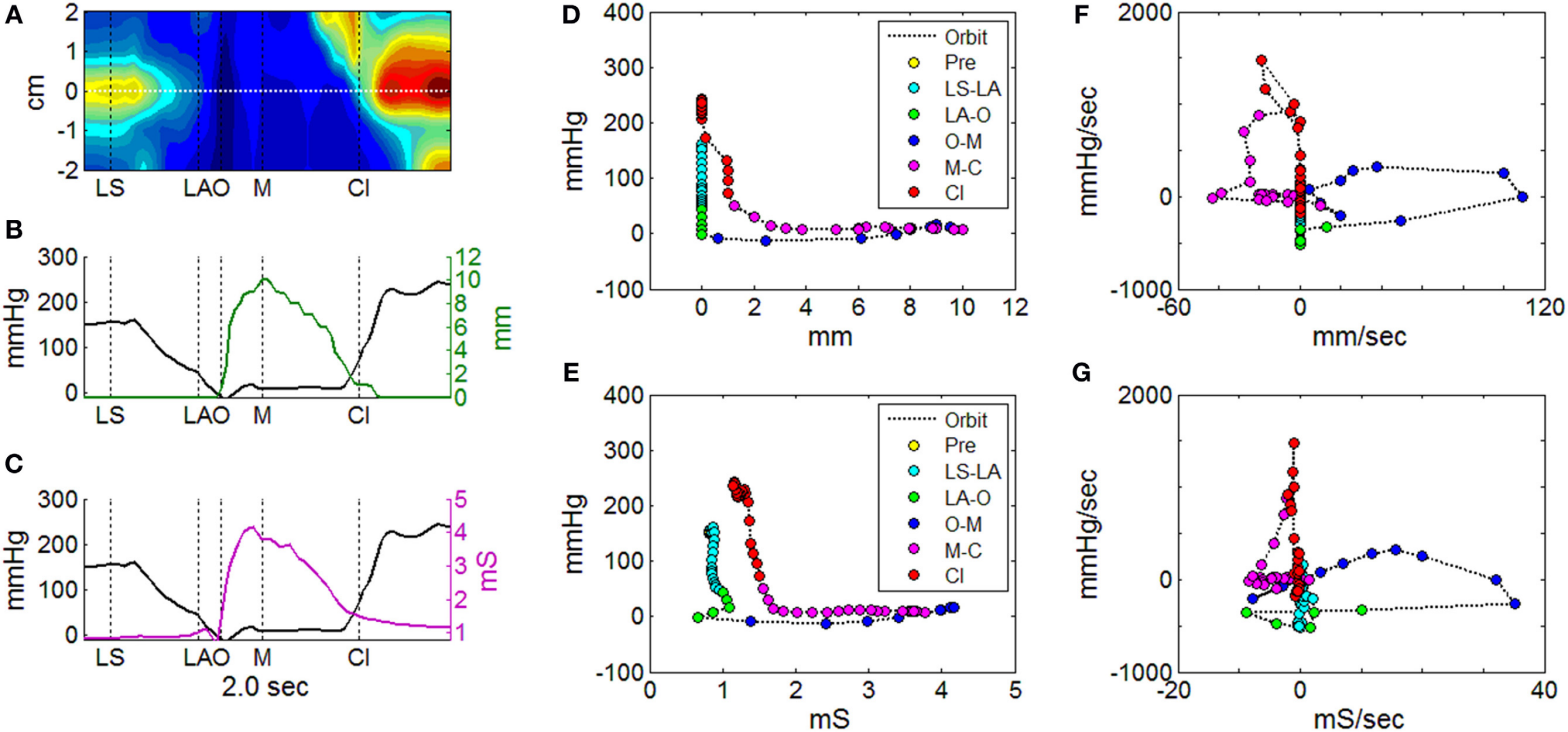
**Orbit plots to describe UES pressure and UES opening over the time of a single swallow**. **(A)** Spatio-temporal plot of pressures recorded during UES relaxation and opening. Horizontal white line indicates the midpoint position of the reference impedance segment over which average diameter was calculated. Vertical black lines indicate timings of important radiological features; onset of laryngeal superior movement (LS), onset of laryngeal anterior movement (LA), onset of UES opening (O), maximum UES diameter (M), and UES closure (Cl). **(B)** Plot of pressure (at position 0 cm in **A**) and mean diameter (based on fluoroscopy) at the reference segment. **(C)** Plot of pressure (at position 0 cm in **A**) and admittance at the reference segment. **(D)** Orbit plot based on diameter vs. pressure data over time in **(B)**, with samples color coded for order of time sequence. **(E)** Orbit plot based on admittance vs. pressure data over time in **(C)**, with samples color coded for order of time sequence. **(F)** Time derivative orbit plot of diameter vs. pressure with samples color coded for time sequence. **(G)** Time derivative orbit plot of admittance vs. pressure with samples color coded for time sequence.

**Figure 8:**
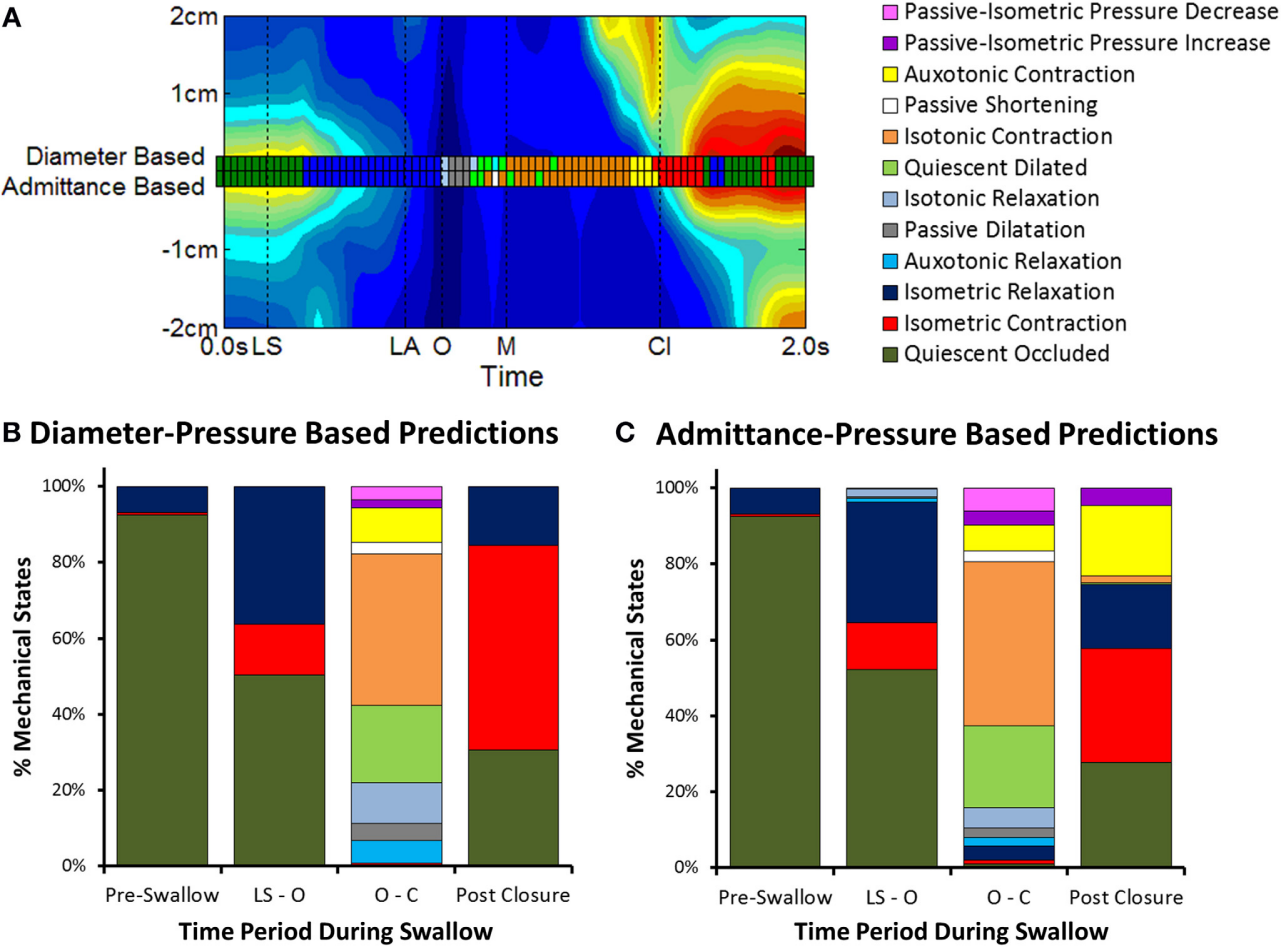
**Changes in mechanical states over time**. **(A)** Comparison of UES mechanical states determined over time for an individual swallow (example swallow in Figure 7A). A separate series of mechanical states are shown superimposed on a spatio-temporal plot of UES relaxation (colored squares) based on the relationships of diameter-pressure and admittance-pressure. Vertical black lines indicate timings of important radiological features; onset of laryngeal superior movement (LS), onset of laryngeal anterior movement (LA), onset of UES opening (O), maximum UES diameter (M), and UES closure (Cl). **(B)** Distribution of mechanical states as calculated by the diameter-based method (gold standard). Columns show data grouped in relation to the different phases of the swallow sequence pre-swallow, the period from laryngeal superior movement to UES opening (LS—O), period from UES opening to closure (O—Cl) and post-closure. **(C)** Distribution of mechanical states predicted by the admittance-based method.

UES mechanical states were defined per Figure 2 using a decision tree (Figure 6). Example orbit plots which describe UES pressure and UES opening over time are shown in Figure 7. The occurrence of mechanical states over time is shown for an example swallow in Figure 8A and the overall prevalence of the different states recorded during all swallows is shown in Figure 8B. The equivalent mechanical states predicted using the admittance-pressure model are also shown for the example swallow and all swallows (Figures 8A,C).

Prior to initiation of a swallow, Occluded Quiescence (reflecting static basal tone of the UES) was the most predominant mechanical state. After initiation of swallow, the proportion of Occluded Quiescence was reduced in concert with increased Isometric Contraction and/or Isometric Relaxation. The Auxotonic mechanical states were typically associated with the onset of UES opening and UES closure on radiology. The period of UES opening ranged from (0.43 to 0.83 s, mean 0.63 s) and correlated with the sum of the predominant states defined by the UES being open (i.e., Passive Dilatation, Isotonic Relaxation, Quiescent Dilatation, Passive Shortening and Isotonic Relaxation; Spearman *r* = 0.629, *p* < 0.05).

When compared to the diameter-pressure predictions (as the gold standard), the admittance-pressure predictions of mechanical states produced similar results (Figure 8C). The overall classification accuracy of the pressure-admittance method for determining mechanical states during the active phases of swallows (i.e., with pre-swallow baseline period excluded) ranged from 62 to 90% (mean 81%). Comparison of graphs in Figures 8B,C shows that the greatest level of discordance between the methods was in relation to states predicted post-luminal closure whereby the admittance-based method classified some Isometric Contraction as Auxotonic Contraction. However, the subject average quantum of time in each mechanical state was not significantly different between methods overall (data not shown). Using the admittance-pressure model, the radiologically defined period of UES opening also correlated with the sum of the predominant states defined by the UES being open (Spearman *r* = 0.586, *p* < 0.05).

#### Modification of the mechanical states model to track superior movement of the larynx

**Figure 9:**
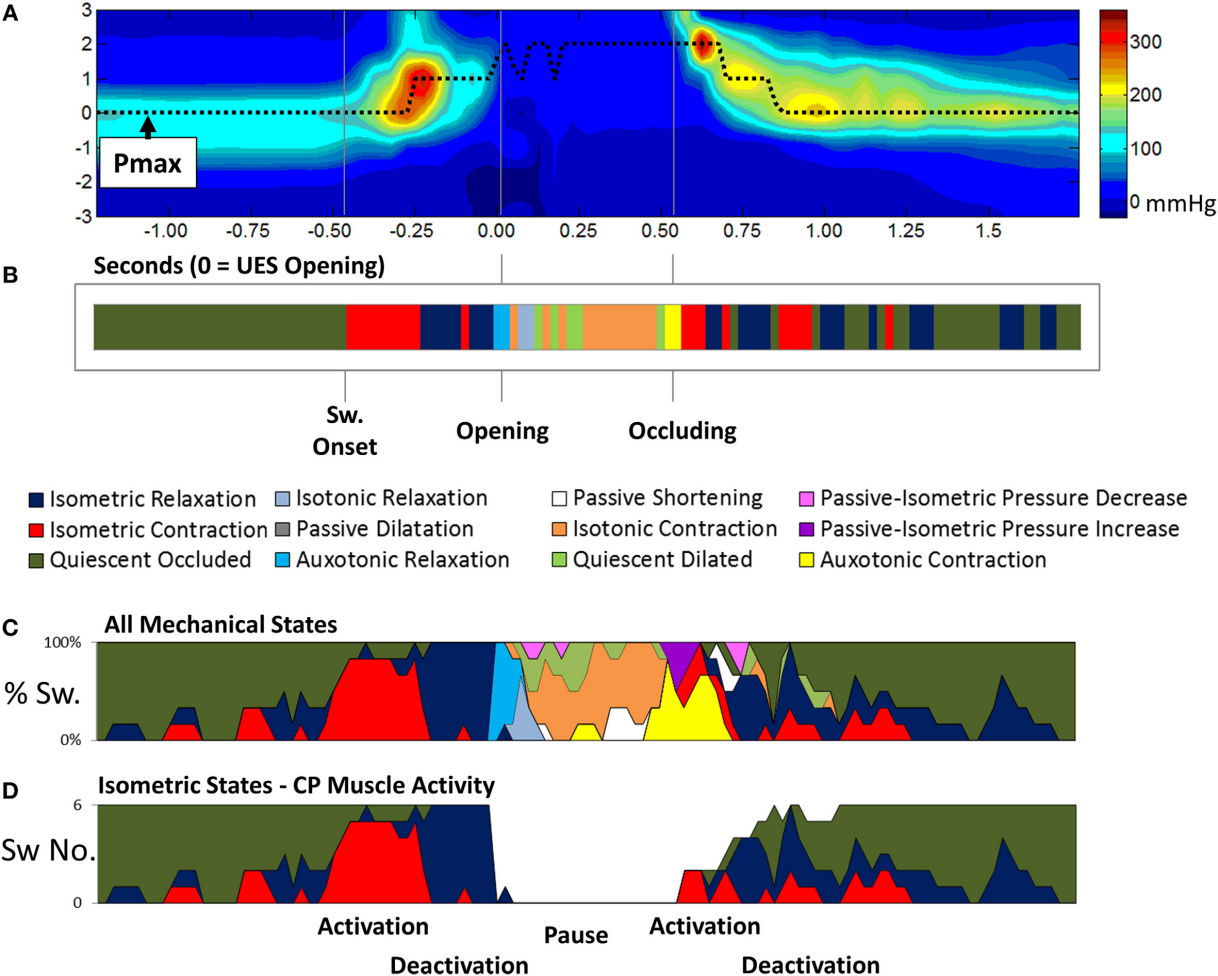
**Adaptation of the pressure-amittance mechanical states model to accommodate movement of the UES high pressure zone relative to the catheter**. **(A)** Spatio-temporal pressure plot of an example pharyngeal swallow showing a region of interest encompassing the UES high pressure zone before, during and after swallow. Pmax shows the positon of maximal pressure defined at all time points and demonstrates the elevation of the sphincter that occurs before complete relaxation. **(B)** A representation of the UES mechanical states predicted during the swallow over time based on the pressure and admittance values determined along the Pmax line. Different phases of the swallow shown are indicated based on the mechanical states predicted. **(C)** Aggregate data from all liquid swallows recorded in this subject showing changes in the proportion of the mechanical states over time. **(D)** The inferred level of activation of cricopharyngeal muscle over time.

With the optimal settings defining the pressure-admittance model determined, the analysis algorithm was modified to accommodate superior axial movement of the UES high pressure zone. This was done using the time and position of maximum pressure (Pmax) recorded within a region of interest large enough to encompass the ~2 cm superior movement of the larynx (Figure 9A). Admittance and pressure were analyzed as before to generate orbit plots and predict mechanical states occurring over time during each individual swallow (Figure 9B). Area plots of the relative proportions of mechanical states over time can be created based upon multiple repeat swallows (Figure 9C). The mechanical predictions allow direct inference of the activity specific to different actions occurring during the swallow. For example activation and deactivation of the CP muscle in isolation based on identification of Isometric Contraction and Relaxation (Figure 9D).

### Studies in asymptomatic controls and motor neuron disease patients

Swallows from 11 MND patients (age 58–91 years, mean 69 years) were compared with 67 Control Subjects. Thirty five Controls were within the same age range as the MND patients and were defined separately as “Age Matched Controls” (age 59–91 years, mean 74 years), the remaining 32 control subjects were of younger age and defined separately as “Younger Controls,” age 20–57 years, mean 38 years. A total of 383 × 5 ml and 370 × 10 ml liquid bolus swallows were analyzed and per-volume averages calculated for each Patient/Subject.

#### Temporal distribution of UES mechanical states during control swallows

**Figure 10:**
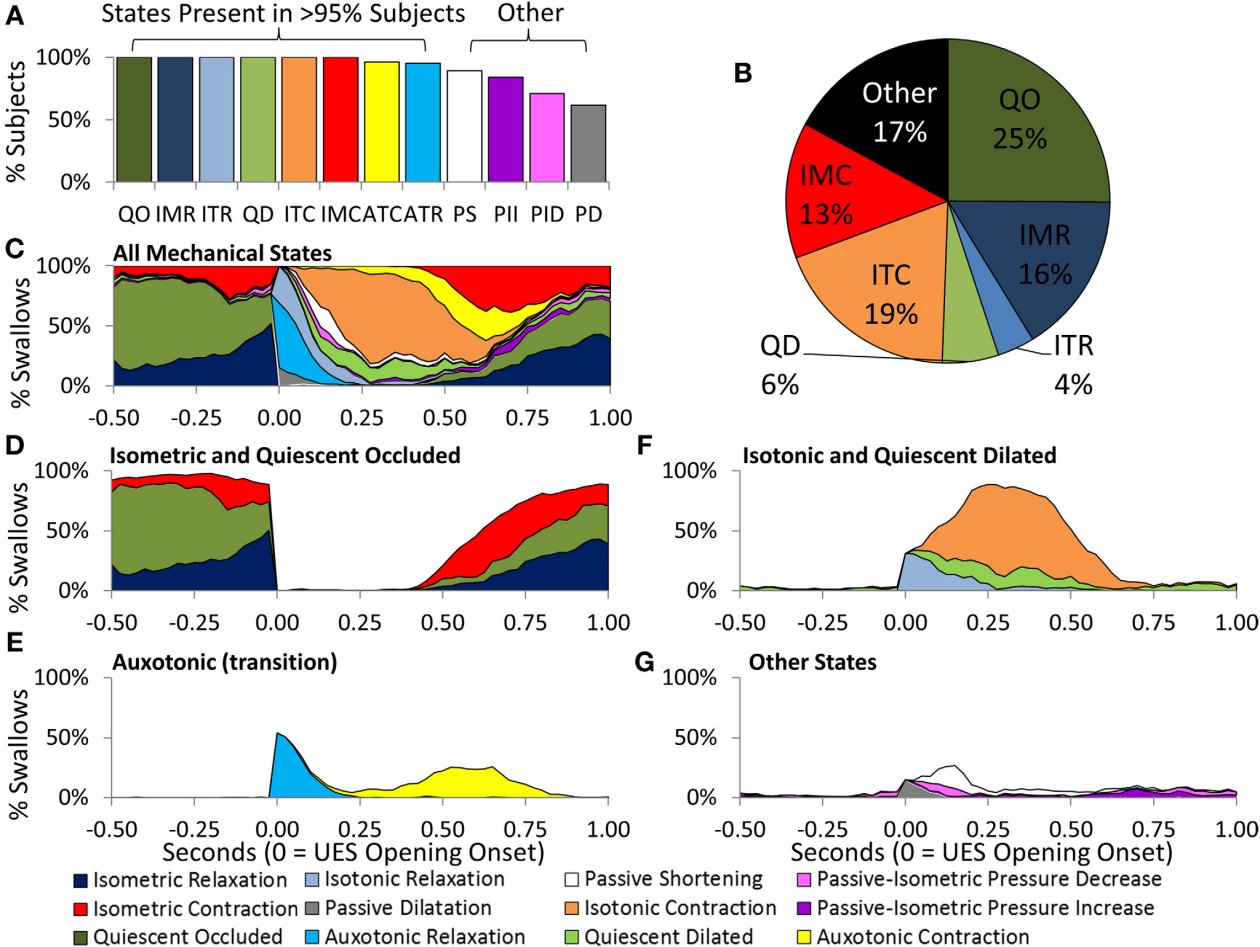
**Distribution of Mechanical States predicted across Control Subjects and in relation to time during 5 ml swallows**. **(A)** The proportion of subjects demonstrating each of the 12 mechanical states. **(B)** Pie chart showing the prevelance of the main mechanical states. **(C)** The timing of occurrence of all mechanical states. **(D)** Isometric States are indicative of CP muscle activation. **(E)** Auxotonic states marked the timing of onset of opening and UES closure. **(F,G)** Timing of remaining states. Data were temporally aligned based on the onset of appearance of the first mechanical state consistent with the luminal being open.

Eight of the 12 predicted UES mechanical states were demonstrated in >95% of Control Subjects and were therefore considered ubiquitous of healthy swallowing (Figure 10A). Four mechanical states predominated overall. These were, Quiescent Occluded, Isometric Contraction, Isometric Relaxation and Isotonic Contraction (Figure 10B). The timing of occurrence of the different mechanical states during the swallows is shown in Figures 10C–G. Isometric States (Figure 10D), indicative of CP muscle activity, were observed independently of Isotonic states (Figure 10F), indicative of the UES opening and closing due to anterior/posterior laryngeal movement. Auxotonic states marked the transition between Isometric and Isotonic states (Figure 10E) and, as previously described, are indicative of the timing of onset of opening and UES closure (see also Figures 9B,C). A similar pattern of mechanical states was observed for both 5 and 10 ml boluses. However, increasing the bolus volume was associated with a longer period of Isotonic Relaxation (5 ml 0.05 s [0.04, 0.07] vs. 10 ml 0.06 s [0.03, 0.08], *p* < 0.05) and Isotonic Contraction (5 ml 0.28 s [0.22, 0.33] vs. 0.32 [0.26, 0.37], *p* < 0.001) as well as a shorter period of Quiescent Occlusion (5 ml 0.34 s [0.23, 0.48] vs. 0.30 s [0.22, 0.42], *p* < 0.01). The sum time in the predominant states defined by the UES being open (i.e., Passive Dilatation, Isotonic Relaxation, Quiescent Dilatation, Passive Shortening, and Isotonic Relaxation) was longer for larger boluses (5 ml 0.45 s [0.38, 0.56] vs. 10 ml 0.50 s [0.42, 0.58], *p* < 0.001).

#### Controls vs. MND patients: UES pressure profile

**Figure 11:**
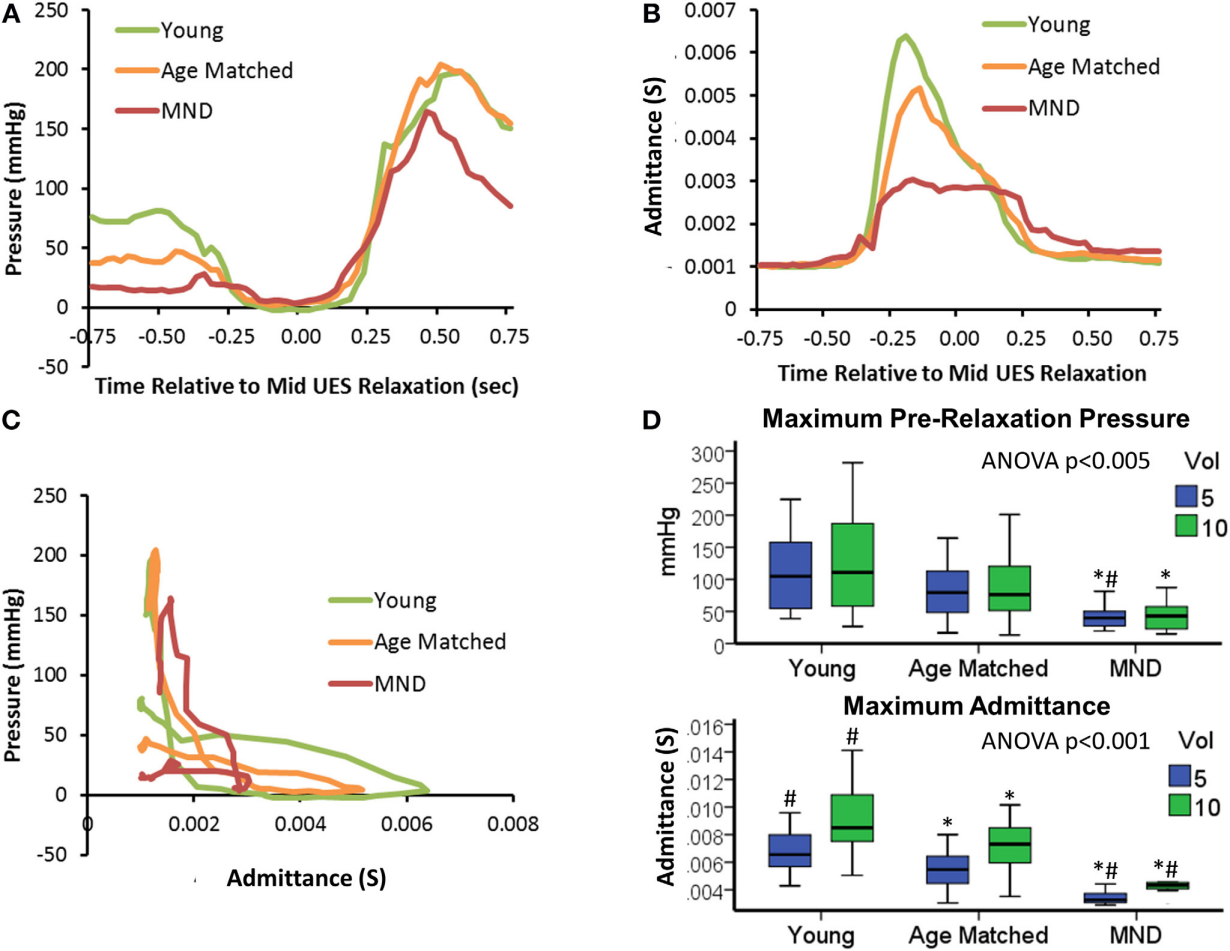
**UES pressure-admittance profiles across study groups**. **(A)** Median UES pressure over time for 5 ml boluses. **(B)** Median UES admittance over time for 5 ml boluses. **(C)** Orbit plots based on admittance and pressure for 5 ml boluses. **(D)** Box plots of maximum pre-relaxation UESpressure and maximum admittance during UES relaxation in relation to groups and bolus volume. ^*^#p < 0.05 vs. ^*^Young, or #Aged Matched for 5 or 10 ml volume.

Profiles of UES pressure recorded during swallowing demonstrated an overall pattern of lower pressures (i.e., weak contractile vigor) with age and MND pathology (Figure 11A). UES pressures recorded pre-relaxation were significantly lower in MND patients (Figure 11D). UES nadir pressures recorded during relaxation were higher in MND for 10 ml boluses vs. Young, but not higher vs. Age Matched Controls (YC −1 [−2, 2], AMC 3 [−1, 8] and MND 4 [1, 9] mmHg, ANOVA *p* = 0.003, pairwise *p* < 0.05 MND vs. YC). The 0.2 s UES integrated relaxation pressure was not significantly different between Groups (5 ml YC −3 [−3, 1], AMC 2 [−2, 8] and MND 4 [1, 7] mmHg, ANOVA *p* = 0.011, pairwise not significant; 10 ml ANOVA *p* = 0.013, pairwise not significant). Post-relaxation peak UES pressures were significantly lower in MND, however only for 10 ml boluses (YC 220 [173, 374], AMC 227 [172, 285] and MND 142 [121, 195] mmHg, ANOVA *p* = 0.006, pairwise *p* < 0.05 MND vs. YC and vs. AMC). Bolus volume had no effect on pre-swallow UES pressure (5 ml 81 [48, 132] vs. 10 ml 77 [45, 137] mmHg) or post-UES relaxation peak pressure (5 ml 216 [149, 288] mmHg vs. 10 ml 214 [162, 289] mmHg). UES nadir pressure and 0.2 s UES integrated relaxation pressure were higher with greater volume (respectively 5 ml 0 [−3, 5] vs. 10 ml 1 [−2, 6] mmHg, *p* < 0.05, and 5 ml 0 [−3, 5] vs. 10 ml 2 [−1, 6] mmHg, *p* = 0.052). The volume effects did not achieve statistical significance when assessed for the MND group alone. ROC analysis identified the lower pre-relaxation maximum UES pressure as the UES pressure variable most able to distinguish MND patients from Controls (AUC 5 ml 0.793 and 10 ml 0.761).

#### Controls vs. MND patients: UES admittance profile

Profiles of UES admittance recorded during swallowing demonstrated an overall pattern of lower admittance (i.e., reduced UES diameter) with age and MND pathology (Figure 11B). The maximum UES admittance during UES relaxation was significantly lower in MND Patients (Figure 11D). Age Matched Controls also had lower UES admittance than Young Controls (see Figures 11B,D). UES maximum admittance was significantly higher with greater volume (5 ml 6 [4, 7] vs. 10 ml 8 [6, 9] mS, *p* < 0.001). The volume effect was reduced but still apparent for the MND group alone (*p* = 0.053). Doubling the volume from 5 to 10 ml increased maximum admittance by of 32, 27, and 21% for YC, AMC and MND groups respectively and increased the duration of UES opening by 6, 16, and 20% for YC, AMC and MND groups respectively. Hence, in relative terms, UES diameter increased by less, but was open for longer in MND patients compared to Controls. ROC analysis identified UES maximum admittance as the most predicative of all measured variables for differentiating MND patients from Controls (Admittance AUC 5 ml 0.971 and 10 ml 0.983).

#### Controls vs. MND patients: mechanical state predictions

**Figure 12:**
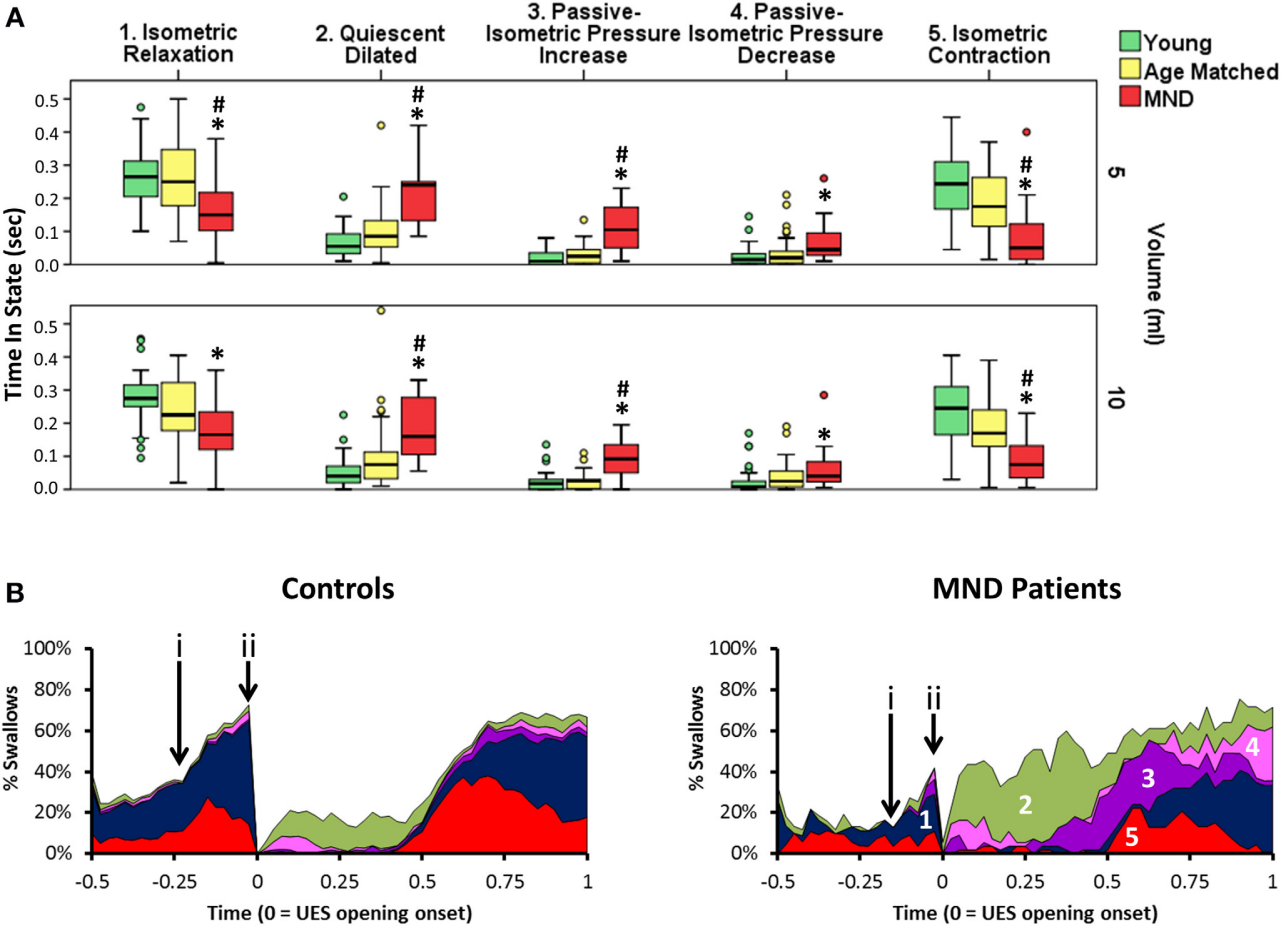
**Predicted UES mechanical states in Controls and Patients with MND**. **(A)** Group differences in relation to the duration of five UES mechanical states that differentiated MND patients from Control Subjects. ^*^# indicates significant One-Way ANOVA and pairwise significance vs. Young Controls^*^ or Age Matched Controls# using Multiple Comparison Procedures. **(B)** Temporal profles of the five mechanical states in panel A as determined for 5 ml swallows. These are numbered in order from 1 to 5 **(A)**. (i & ii) indicate the timepoints of increased activity consistent with swallow onset and luminal opening respectively. Note this suggests that Isometric activity increase is both diminished and delayed.

Orbits constructed by plotting the median UES admittance profile against the median UES pressure profile clearly differentiate the three groups, with substantial attenuation of both admittance and pressure constraining the extent of the orbit of MND patients (Figure 11C). This translated to differences in relation to the UES mechanical states predicted. Based on the average quantum of time during which the different mechanical states were predicted, Isometric States, both Relaxation and Contraction, occurred for a shorter time in patients with MND (Figures 12A,B). The temporal profile of mechanical states (Figure 12B) also suggests that the typical pattern of increased Isometric Activity consistent with swallow onset (as described in Figure 9D) is both diminished and delayed in MND patients compared to controls. The Quiescent Dilated state was present for a longer time in MND patients (Figure 12A). The total time in UES open states was not significantly different between MND patients and Age Matched Controls (AMC 0.53 s [0.44, 0.64] and MND 0.62 s [0.49, 0.74]).

Amongst the mechanical states that were not ubiquitous for Healthy Subjects, we observed a two-fold greater amount of Passive-Isometric Pressure Increase in MND patients compared to both Young and Age Matched Controls. This observation was consistent for both bolus volumes (Figure 12A). Passive-Isometric Pressure Decrease was also more common in MND, however only statistically significant when compared to Young Controls (Figure 12A). Figure 12B shows the temporal profiles and overall distribution of the five Mechanical States which differentiated MND from Control swallows. ROC analysis identified a longer period Passive-Isometric Pressure Increase as the UES mechanical state most predicative for differentiating MND patients from Controls (AUC 5 ml 0.879 and 10 ml 0.904).

#### Controls vs. MND patients: pressure and admittance change during mechanical states

**Figure 13:**
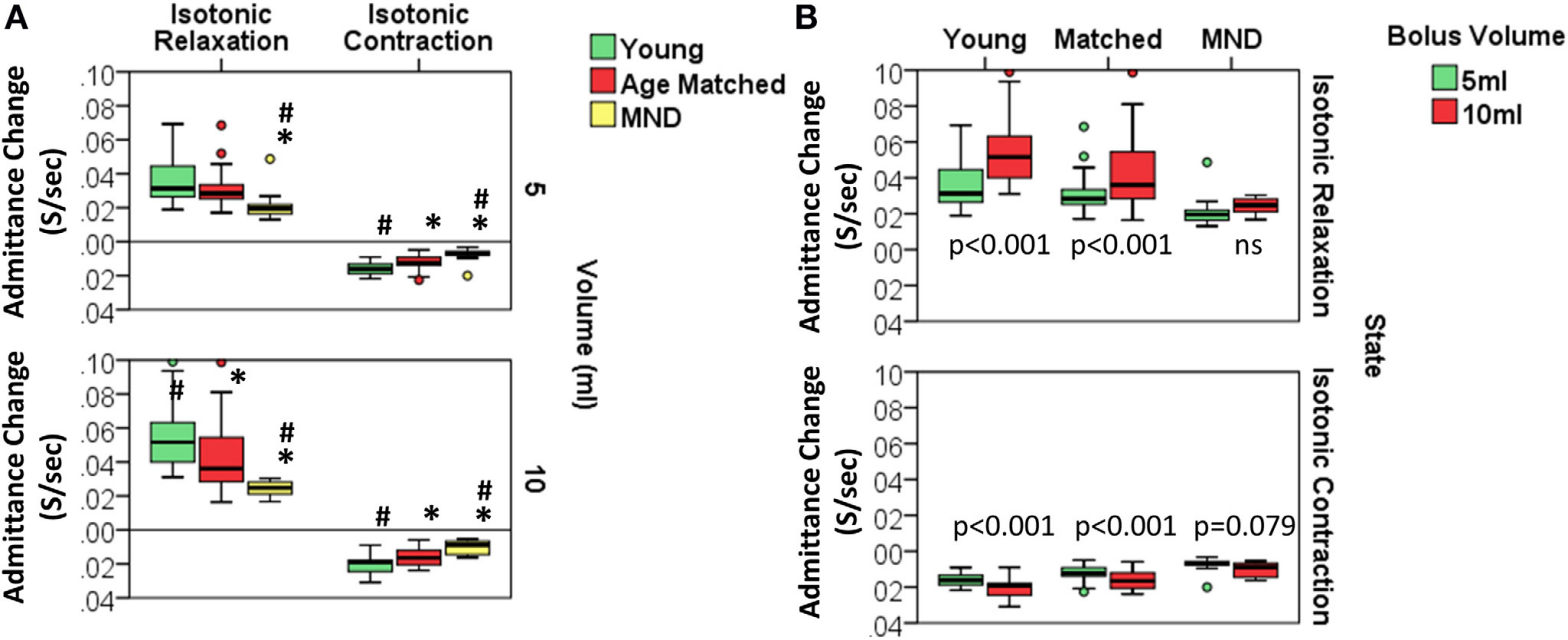
**Rate of admitance change during Isotonic UES mechanical states**. **(A)** Group comparisons for each volume. ^*^# indicates significant One-Way ANOVA for uniform volume and pairwise significance vs. YC^*^ and/or AMC# using Multiple Comparison Procedures. **(B)** Comparisons in relation to bolus volume. Paired *t*-test *p*-values are shown.

Further analyses were performed to examine differences in the rate of pressure and rate of admittance increase or decrease in relation to the different mechanical states. For Isometric States, defined by an increasing or decreasing lumen occluded pressure, the rate of pressure increase or decrease was not significantly different. The volume swallowed also had no effect on the rate of pressure change. For Isotonic States, defined by the lumen opening or closing, the rate of admittance increase or decrease was significantly altered in MND patients where a slower rate of admittance change was observed indicating a slower rate of UES opening and closure (Figure 13A). Furthermore, older Age-Matched Controls had a diminished rate of admittance increase or decrease when compared to Younger Controls. A significant volume effect was also observed in Controls whereby the rate of admittance increase or decrease during Isotonic Contraction or Relaxation was more rapid in relation to the larger 10 ml boluses (Figure 13B). In contrast MND patients did not show a significant effect of volume on the rate of admittance change during Isotonic States (Figure 13B).

## Discussion

The aim of this study was to validate and apply a novel method to objectively define the mechanisms that contribute to deglutitive UES relaxation and opening. A “mechanical states” method, previously developed for use in *ex vivo* gut preparations, was adapted to allow prediction of mechanical changes occurring *in vivo* within the UES during normal and disordered liquid bolus swallowing. The significant advantage of this method is that it not only gives a more complete description of the mechanical state of the muscle but potentially enables the likely neural inputs that generate these to be deduced. The main study findings were (1) admittance change was a sufficiently good correlate of diameter change to allow mechanical states to be predicted for the UES *in vivo* and without the need for simultaneous radiological imaging; (2) of 12 theoretically possible UES mechanical states, eight are nearly always seen during healthy swallowing; (3) a pattern of increase, decrease and pause in “Isometric” UES mechanical states was seen and this was consistent with known neurally dependent phasic discharge patterns of CP muscle EMG activity during swallowing, (4) “Auxotonic” mechanical states marked the transition to the UES being open or closed, (5) the presence of combined “Isotonic,” “Passive,” and “Dilated” states defined the period of UES opening, (6) the duration of “Isotonic” states, as well as rate of admittance change seen, increased with larger bolus volumes in concert with adaptive accommodation of the UES opening, (7) “Passive-Isometric Pressure” states were a pathological manifestation rarely seen in healthy individuals, and finally (8) UES maximum admittance was a better non-specific marker of progressive swallowing dysfunction related to age and the presence of motor neuron disease, than UES pressure recorded before, during or after UES opening. Mechanical states revealed in this study are consistent with the known neural inputs activating the different muscles during swallowing and some indicate more details of the mechanical consequences that result when these complex neuromuscular processes become aberrant due to pathology.

Methodology to better assess UES function is clinically relevant for diagnosis of motor dysfunctions causal of dysphagia symptoms. Appropriately diagnosed UES dysfunctions may be treatable through interventions such as dilatation (Hatlebakk et al., 1998; Clary et al., 2011; Lan et al., 2013), Botox injection (Kelly et al., 2013), surgical intervention (e.g., myotomy) to ablate cricopharyngeal tone (Mason et al., 1998; Kelly, 2000) or swallow manoeuvrers and exercise. For example, the Mendelsohn maneuver to augment UES opening (Kahrilas et al., 1991) and the Shaker suprahyoid muscle strengthening exercise (Shaker et al., 1997, 2002).

Traditionally, the manometric assessment of UES function has been based upon quantification of hypopharyngeal intrabolus pressure, as a marker of UES flow resistance, and UES residual pressure, as a marker of extent of UES relaxation. However, intrabolus pressure is only a predictor of UES flow resistance when measured in conjunction with an *intact* pharyngeal swallow and UES residual pressure does not equate to UES opening diameter, such that a fully relaxed UES may nevertheless not open (Williams et al., 2002). In radiotherapy induced dysphagia the CP muscle can have poor contractile tone due to reduced muscle fibre density, whilst at the same time displaying restricted opening and poor compliance due to interstitial fibrosis. An important distinction between the traditional approach and the approach we have applied in the current study is that our assessments are based on direct simultaneous measurements of both UES pressure and UES opening. In our study we used videofluoroscopy as the “gold standard” to measure UES opening and closure. As applied, this provided only a single lateral projection of the UES and therefore could only be considered accurate in this one dimension. Whilst the largest changes in UES diameter do occur in this anterior-posterior dimension, without 3D cross sectional imaging, our measurements are only a good approximation of the real volume change that occurs within the UES region during swallowing. Nevertheless, by measuring both pressure and opening we aimed to derive novel physiological markers which could provide a more complete assessment of swallowing pathophysiology.

The prediction of mechanical states based on the relationships of diameter change and pressure change is a novel concept that was originally applied to the physiology of gut motility and has further elucidated the neuro-mechanical basis of peristalsis (Costa et al., 2013; Wiklendt et al., 2013; Dinning et al., 2014). In the current study we have applied this same novel approach to the assessment of UES mechanical function *in vivo*. The physiology governing UES relaxation and then opening is very different to gut peristalsis, however the fundamental mechanical principles governing propulsion of contents are essentially the same. That is, the lumen ahead of the moving bolus must relax and open to allow unimpeded passage and the lumen behind the bolus must contract and close to generate propulsive force and prevent retrograde bolus escape (Dinning et al., 2014).

In relation to UES opening during deglutition, past studies have shown that the CP muscle is the major contractile element of the UES which often contracts at the onset of swallow (in concert with superior laryngeal movement) and then relaxes prior to being opened by traction forces applied by the anterior movement of the hyoid and larynx (Asoh and Goyal, 1978; Cook et al., 1989; Clary et al., 2011). UES “relaxation” is a manometric term used to describe the reduction in CP muscle activity which precedes UES opening. The onset of UES relaxation often follows a brief “fore-burst” of myogenic activity, which occurs in concert with laryngeal adductor activity. UES relaxation is then followed by a pause in activity whilst the UES is open (Asoh and Goyal, 1978; Lang et al., 1991; Ertekin et al., 1995; Jones et al., 2014). A central pattern generator controlled burst of EMG activity then follows luminal closure leading to a post-relaxation pressure peak.

As demonstrated in the current study (Figure 9D), the mechanical states model allows lumen occluded pressures to be differentiated into distinct periods of Isometric Contraction and Isometric Relaxation. Our measurements show that the onset of swallowing is associated with an increased prediction of Isometric Contraction states followed sequentially by Isometric Relaxation states, a pause in Isometric States and then a post-relaxation increase in Isometric Contraction states. These observations are highly consistent with previous EMG studies which have directly measured activation and deactivation of the CP muscle during swallowing (Asoh and Goyal, 1978; Van Overbeek et al., 1985; Lang et al., 1991; Medda et al., 1997; Jones et al., 2014). Our observations show that pressure-impedance based predictions of mechanical states may potentially determine the level CP muscle activity. However, direct correlation of CP EMG activity and pressure-diameter based predictions of the Isometric mechanical states is still needed to confirm this. Given that CP muscle EMG is less applicable to the clinical setting, while high resolution impedance-manometry is already in widespread use, our method may have clinical relevance if proven to be a direct correlate of CP muscle activation.

In healthy individuals, UES opening demonstrates an adaptive response to bolus volume as a consequence of sensory feedback mechanisms. Hence swallowing of larger boluses is accommodated by earlier and larger increases in UES diameter (Kahrilas et al., 1988; Cook et al., 1989). The net effect of this adaptive response is the ability of the pharyngo-esophageal segment to accommodate a faster rate of bolus flow without any increase in flow resistance. In the current study we observed several effects which elucidate this adaptive response to a larger bolus volume. These were; (1) a wider diameter based on an increase in maximum admittance; (2) a longer UES opening period based on the sum predicted time in mechanical states defined by the lumen being open; and (3) a faster rate of UES opening indicated by the rate of admittance change during Isotonic Relaxation. In contrast our results show no effect of bolus volume on the UES pressures before or after UES relaxation, a finding which is consistent with past studies that show that pharyngeal and UES contraction is far less adaptive, following a more stereotypical constant contractile pattern (Cook et al., 1989).

Nadir UES pressure and UES integrated relaxation pressure were higher with larger volumes, a finding which is consistent with intrabolus pressure being a factor in maintaining the UES in an open state (Cook et al., 1989; Pal et al., 2003). Our radiological validation studies showed that three mechanical states were predicted to occur at the onset of radiological UES opening, namely Auxotonic Relaxation, Passive Dilatation and Isotonic Relaxation. Of these, the state of Passive Dilatation is the only state that is mechanically consistent with UES opening being facilitated by bolus pressurization from above. Across study subjects, Passive Dilatation was by far the least frequently observed mechanical state suggesting that, in health, intrabolus pressures are of minimal importance for initiation of UES opening. Previous videomanometry studies have concluded the same, showing that anterior hyoid movement initiates opening after which bolus forces determine the extent of opening (Cook et al., 1989).

Analysis of 5 and 10 ml bolus swallows in healthy controls demonstrated minimal age group related effects on pressure measurements. UES contractile pressures were numerically lower and UES nadir pressures were numerically higher in the older age group, however these differences were not statistically significant. The main age-related effects observed were in relation to UES pre-relaxation pressure and UES admittance. Older healthy subjects demonstrated a similar duration of UES relaxation and opening to younger subjects, but had a lower pre-relaxation pressure and lower maximum admittance during opening. Lower pre-relaxation pressures suggest that passive-elastic forces and basal myogenic and neurogenic activity of the CP muscle may be reduced with aging. In contrast, post-relaxation peak pressures were similar between young and aged subjects, suggesting no age-related effect on the pattern-generator mediated post-relaxation rebound burst CP muscle activity. Lower UES admittance in aged subjects suggest a reduced UES diameter which is supported by past radiological studies and possibly due to an age-related reduction in UES compliance (Shaw et al., 1995).

Our measurements also suggest that the *rate* of diameter change during the *early* (Isotonic) phase of UES opening was slower in aged subjects. Reduced compliance of the CP muscle and adjacent structures may explain this observation. An alternative explanation is that the traction forces being applied by the hyoid muscles at the onset of UES opening are weaker in the aged. Hence the mechanical distraction of the UES may occur at a slower rate due to a combination of both decreased UES compliance and/or muscle weakness. Suprahyoid strengthening exercises (Shaker exercise) are known to improve UES opening in aged subjects (Shaker et al., 1997). In light of our observations, the beneficial effects of the Shaker exercise may relate to either re-training of weakened muscles to open a normally compliant UES, or augmentation of normal muscles in order to open a less compliant UES.

MND patients showed marked deterioration in their swallowing function. Interestingly, the differences seen between MND patients and Age-Matched Controls were of a similar, but more severe, pattern to the subtle differences seen between Age-Matched and Young Controls. Hence the changes documented across the groups suggest a continuum of progression of a similar overall pattern of change in motor function. Whilst the healthy Aged can still swallow effectively, they retain less functional reserve, meaning that they will develop swallowing difficulties earlier should a disease process, such as MND, supervene.

Compared to Age-Matched Controls, UES contractile pressures were lower and UES nadir pressures were slightly but significantly higher in MND. Mechanical state predictions suggested that MND patients have significantly diminished Isometric activity which we have interpreted as being consistent with diminished neurogenic activation of the CP muscle. However, in order to confirm this, further studies are needed to correlate these findings with simultaneous EMG recordings. Consistent with a past videofluoroscopy study (Higo et al., 2004), our measurements suggest that duration of UES opening was similar, or possibly longer, in MND vs. Age-Matched Controls. However we also observed a lower UES admittance and slower admittance change in MND patients. Hence the rate and extent of UES opening was diminished in MND. These findings are consistent with the attenuation of force generation capacity of the hyoid muscles. The fact that volume related changes were still observed in MND despite substantial mechanical weakness suggests that the sensory coding for volume may still be preserved.

**Figure 14:**
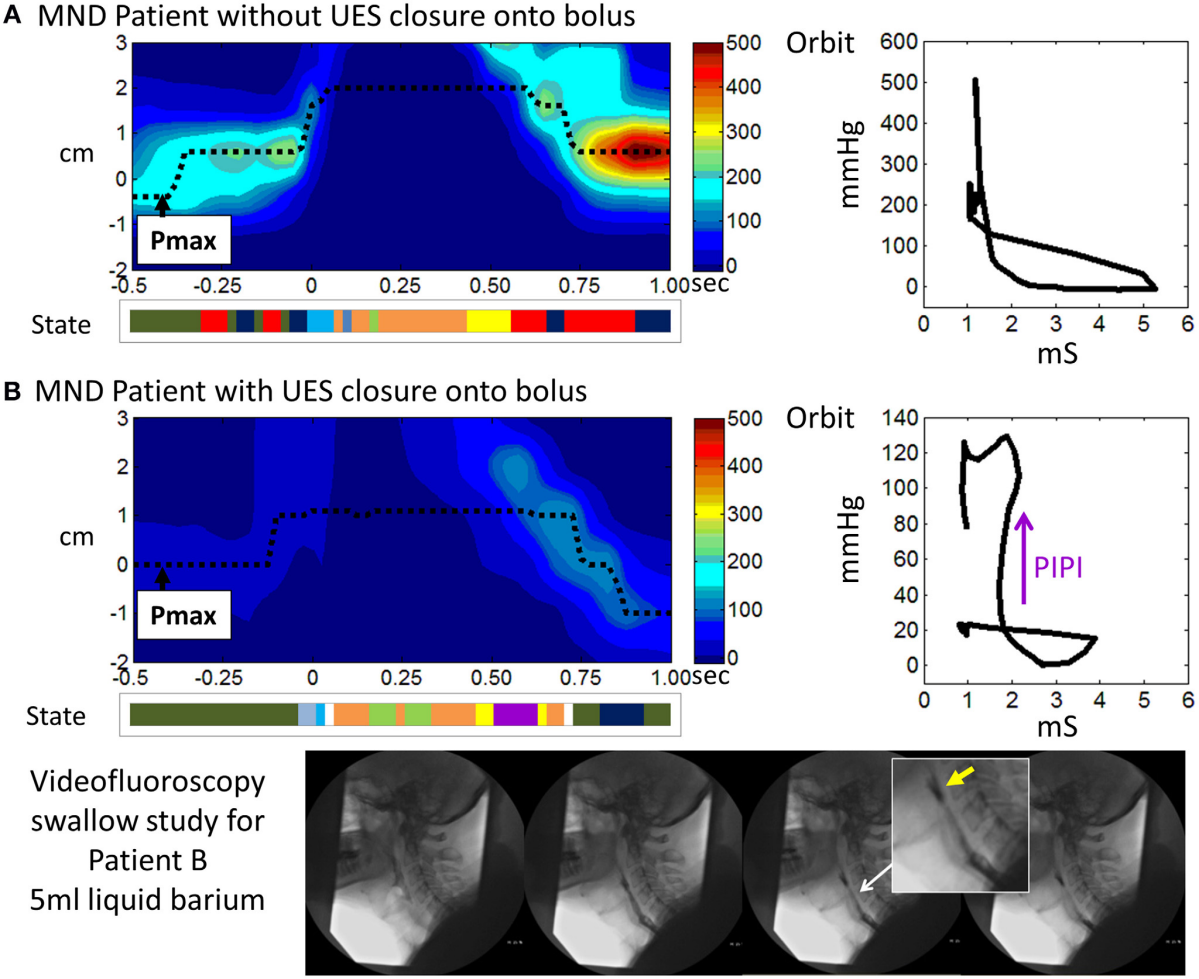
**Illustrative examples from MND patients**. **(A)** An early stage MND patient (Female 69 years) who still swallows well. **(B)** A patient with more advanced MND (Male 62 years) in whom the mechanical state of Passive-Isometric Pressure Increase (PIPI) is predicted by the model to occur with luminal closure. Spatio-temporal pressure plots show the pressures of UES high pressure zone during a 5 ml liquid swallow. Pmax defines the position of maximum axial pressure. A representation of the UES mechanical states predicted for the swallows is shown below each plot (color scheme identical to previous Figures, PIPI in colored purple). Admittance-pressure Orbit plots for each swallow are shown right. Videofluoroscopy images from a barium swallow investigation of Patient B performed within 1 week of the high resolution impedance manometry study are shown at the bottom of the figure. The images captured during 5 ml liquid barium swallow show UES closure onto, rather than behind, the swallowed bolus. Hence the bolus is divided into two as the UES closes and bolus is retained in the pharynx above the UES as residue (yellow arrow in magnified image). Residual bolus material is most likely also trapped within the UES lumen.

The mechanical state Passive-Isometric Pressure Increase was significantly augmented in the MND patients. Passive-Isometric Pressure States were only rarely observed during healthy swallowing. Hence emergence in MND suggests a marker of disease severity in relation to its impact on swallowing. Moreover, the prediction of Passive-Isometric Pressure Increase suggests UES contraction when the lumen still contains swallowed bolus. This pattern, when seen on clinical videofluoroscopy, is often described as “premature UES closure.” However, our data suggest that UES opening in our MND patients differed mostly with respect to the rate and extent of opening, rather than the duration of opening. We therefore conclude that Passive-Isometric Pressure Increase is seen because the swallowed bolus fails to fully transit the UES region and therefore the UES contracts onto, rather than behind, the bolus. Hence, rather than the bolus being fully propelled distally when the UES closes, some residual bolus material may be trapped within the UES lumen, which would be consistent with catheter based measurement of higher UES admittance and the definition of Passive Isometric Pressure, rather than Isometric Contraction (see patient examples provided in Figure 14). Delayed bolus transit through the pharynx has been previously reported in MND patients using videofluoroscopy (Higo et al., 2004), scintigraphy (Fattori et al., 2006) and fiber optic endoscopic evaluation of swallowing (D'Ottaviano et al., 2013). A slower rate of transit is most likely due to weak lingual propulsion in combination with weak pharyngeal pressure generation and a reduced UES aperture.

Some EMG measurements in MND patients show premature UES closure due to augmented CP activity and a short period of CP pause (Ertekin et al., 1995, 2000). These observations suggest a paradoxically enhanced, yet uncoordinated, UES myogenic activity in MND in a setting of diminished central pattern generator mediated activity overall. In contrast, our findings showed attenuated Isometric activity with UES opening of normal duration but restricted aperture. The fact that evidence for paradoxically augmented CP activity was not clearly demonstrated in our MND patients may suggest a lack of detection sensitivity of the mechanical states method or that there are differences in relation to the specific MND sub-pathology of the patients' we studied. Simultaneous EMG studies would be needed to confirm whether the previously reported CP activity was absent in our patients, or present, but undetected by our method.

In conclusion, we present findings in relation to a novel method to assess UES function using the prediction of UES mechanical states based on the relationship of admittance and pressure. Changes in mechanical states in health emulate the established understanding of UES opening mechanics as previously determined using videomanometry. Mechanical state predictions were simple to apply using software and revealed patterns consistent with the known neural inputs activating the different muscles during swallowing. Future simplification of the analysis and key interpretations will allow this method to be more readily applied. Further validation studies are needed to correlate mechanical states predictions with EMG recordings and to determine UES mechanical states in patients with obstructive pathologies.

## Author contributions

Taher I. Omari, Philip Dinning, Lukasz Wiklendt, and Charles Cock; study concept and design, analysis and interpretation of data, draft and critical review of manuscript. Nathalie Rommel and Marcello Costa critical manuscript review and interpretation of data.

### Conflict of interest statement

The authors declare that the research was conducted in the absence of any commercial or financial relationships that could be construed as a potential conflict of interest.
